# Lipidomic Analysis of Human Plasma and Hippocampus Across Alzheimer’s Progression and Preclinical 5xFAD Mouse Model

**DOI:** 10.1007/s12035-026-05849-1

**Published:** 2026-04-13

**Authors:** Enrico Castroflorio, Joan Cabot, Marc Miralles, Margalida Suau-Fullana, María Peter, Gabor Balogh, Zsolt Torok, Eloy Rodríguez, Pascual Sanchez-Juan, Paula Férnandez-García, Victoria Llado, Pablo V. Escribá, Manuel Torres

**Affiliations:** 1 Laboratory of Molecular Cell Biomedicine, Department of Biology, University of the Balearic Islands, Palma, Spain; 2 Department of R&D, Laminar Pharmaceuticals S.A., Palma, Spain; 3 Institute of Biochemistry, HUN-REN Biological Research Centre, Szeged, Hungary; 4 Department of Medicine and Psychiatry (Universidad de Cantabria) and Institute for Research Marqués de Valdecilla (IDIVAL), Neurology Service, Marqués de Valdecilla University Hospital, Santander, Cantabria Spain; 5 Reina Sofia Alzheimer Center, CIEN Foundation, ISCIII, Madrid, Spain; 6 CIBERNED, Network Center for Biomedical Research in Neurodegenerative Diseases, ISCIII, Madrid, Spain

**Keywords:** Alzheimer’s disease, Brain lipids, Plasmalogens, Phospholipids, Sphingolipids

## Abstract

Alzheimer’s disease (AD) poses a significant global health burden, underscoring the need for early and accessible biomarkers to enable timely diagnosis and intervention. Lipids, which constitute over half of the brain’s mass, play essential roles in numerous cellular processes, and their dysregulation has been increasingly implicated in AD pathophysiology. In this study, we performed lipidomic profiling of hippocampal samples derived from individuals at different Braak stages and plasma samples from patients with mild cognitive impairment (MCI), AD, and healthy controls. Parallel analyses were conducted in 5xFAD transgenic mice and wild-type littermates. Our results revealed lipid alterations across central and peripheral compartments in both human subjects and the 5xFAD mouse model. Notably, specific lipid changes identified in particular lipid species at early/mild Braak stages or in MCI persisted into advanced stages of the disease, highlighting the systemic nature of lipid dysregulation in AD and supporting the potential of these lipid signatures as diagnostic and prognostic biomarkers.

**Supplementary Information:**

The online version contains supplementary material available at 10.1007/s12035-026-05849-1.

## Introduction

Dementia is marked by a progressive decline in cognitive abilities, functional independence, and behavioral stability, imposing a substantial societal burden. Globally, Alzheimer’s disease (AD), the leading cause of dementia, is estimated to contribute to annual healthcare costs exceeding €500 billion [1]. As the population ages, the impact of AD and other dementias is expected to increase significantly.

AD is characterized by progressive dementia, memory loss, and impairments in several other cognitive skills [2]. The exact cause of this complex, multifactorial disease remains unresolved. In addition to proteinopathies, factors such as oxidative stress [3], inflammation [4], metabolic disturbances [5], and others contribute to its development. Pathological progression in AD is commonly staged using the Braak classification, which is based on the distribution and severity of neurofibrillary tangles (NFTs) composed of hyperphosphorylated tau protein (tau pathology), ranging from Braak I–II (typically related to preserved cognitive function) to Braak III–IV (mild/moderate AD) and Bra ak V–VI (advanced stages of AD). Concerning a histopathological level, it is unlikely to find lesion-free brain tissue in individuals over 60 years, even if they show no cognitive symptoms. One common finding in cognitively healthy elderly individuals is the presence of NFTs, particularly limited to the entorhinal cortex. This pattern corresponds to Braak stages I–II, which are considered part of normal aging or related to primary age-related tauopathy (PART) [6]. As tau pathology progresses, it spreads to limbic structures (Braak III–IV) and eventually to widespread neocortical areas (Braak V–VI), which are strongly associated with cognitive decline and clinical Alzheimer’s disease [7]. Other lesions frequently found in aged brains include diffuse amyloid-β (Aβ) plaques (they may be neuritic plaques), microinfarcts, gliosis, etc. Importantly, many of these changes may remain clinically silent [8, 9].

Emerging therapies with monoclonal antibodies like lecanemab show promise only in early to mild pathology cases [10]. The absence of effective treatments and the possibility of prevention highlight the urgent need to identify early biomarkers for diagnosing AD. Although AD progresses gradually, from an asymptomatic (or preclinical) stage to mild cognitive impairment (MCI) and eventually to severe cognitive decline and dementia, clinical AD symptoms typically appear only after a long prodromal phase of MCI, during which neuropathological changes, such as neuronal death, are already underway. As a result, diagnosing AD is particularly difficult in the early stages. Therefore, there is a strong demand for identifying peripheral biomarkers that can accurately diagnose AD at an early stage, enabling timely intervention and improving treatment outcomes.

Emerging evidence suggests that pathological changes in AD extend beyond the central nervous system to the peripheral system, raising the potential for non-invasive biomarker detection in blood [11]. Among potential candidates, lipids stand out due to their fundamental roles in brain structure and function. Lipids constitute approximately 50% of the brain’s dry weight, making them highly representative of the brain’s state and function. As essential components of cellular membranes, myelin sheaths, and signaling pathways, lipids play crucial roles in neurotransmission, synaptic plasticity, and overall brain health and homeostasis [12]. Changes in lipid composition or metabolism can significantly impact neurological processes [13–15] and membrane lipid therapy (MLT, melitherapy) has emerged as a promising approach for dementia [16, 17]. Therefore, lipids are considered excellent candidates as biomarkers in blood [18–20]. Consistently, in GWAS studies, many of the genetic alterations recently described in AD have been linked to lipid metabolism, neuroinflammation, and energy metabolism [21, 22].

Several lipidomic studies have investigated Alzheimer’s disease in both human brain tissue and animal models. Among them, Chan et al. [23] performed an extensive comparative lipidomic analysis of over 300 lipid species across different brain regions in late-onset AD patients and in transgenic mouse models, identifying alterations in a variety of lipid species, including sphingolipids, cholesterol esters, and gangliosides. Other studies have primarily focused on peripheral lipid biomarkers, such as phosphatidylcholines and sphingomyelins, in plasma or serum of AD and MCI patients [19, 24]. While these investigations have provided valuable insights into disease-related lipid dysregulation, most did not analyze brain and plasma in parallel, nor did they integrate lipid profiles with neuropathological staging. In this study, we performed lipidomic profiling of hippocampal and plasma samples from both human patients diagnosed with mild cognitive impairment (MCI) or AD (clinical diagnosis) and different Braak stages (histopathological diagnosis). This study was also extended to hippocampus and plasma from a wild-type and 5xFAD mouse model of AD. The primary objective was to identify novel lipid biomarkers in both brain and plasma, and determine whether findings from human samples are mirrored, at least partially, in 5xFAD mice. This analysis aims to support the development of lipid-targeted therapies designed to slow disease progression, which are often tested in transgenic mouse models. Our analysis revealed changes in specific lipid species between the hippocampus and plasma in both humans and mice, suggesting a robust link between central nervous system lipid dysregulation and peripheral biomarkers. These findings highlight the importance of lipid alterations in understanding AD progression and emphasize the systemic dimension of the disease, opening new avenues for the identification of lipid-based biomarkers and therapeutic targets.

## Materials and Methods

The Experimental Procedures in this work abide by the Declaration of Helsinki principles.

### Human Material

Human samples were obtained from national biobanks under authorization by the Committee for Ethical Research of the Balearic Islands (CEI-IB), dependent on the regional Government of the Balearic Islands (GOIB) (Spain). Postmortem human hippocampus samples ranging from 58 years of age to 97 were classified as Braak I–II (*n* = 11), Braak III–IV (*n* = 17), and Braak V–VI (*n* = 20) based on postmortem histopathological diagnosis [2]. These samples were obtained from the Brain Bank of the Institute of Neuropathology of the University Hospital Bellvitge at the IDIBELL (HUB-ICO-IDIBELL-Biobank) (L'Hospitalet de Llobregat, Barcelona, Spain) and the Biobanc-Hospital Clinic -Institut d’Investigacions Biomediques August Pi I Sunyer (IDIBAPS) (Barcelona, Spain). Briefly, Braak staging is a neuropathological system that classifies the progression of tau-related neurofibrillary pathology in Alzheimer’s disease from early to late involvement of brain regions. It describes six stages (I–VI): starting in the entorhinal cortex and related areas (I–II), then spreading to the hippocampus and limbic regions (III–IV), and finally reaching neocortical areas (V–VI). Higher Braak stages reflect greater anatomical spread of tau pathology and typically correspond to more advanced cognitive decline. In contrast, early Braak stages (I–II) usually correspond to cognitively unimpaired (no AD or low probability according to Hyman et al. [25]), although cognitive alterations related to other neurological disorders should not be discarded. Braak I–II samples are used in this work as neuropathological controls, and Braak III–IV (mild/moderate AD) and V–VI (advanced AD) groups were compared with the reference group Braak I–II [26].

Plasma samples ranging from 52 years of age to 77 from cognitive normal controls (*n* = 7), mild cognitive impairment (*n* = 6), and Alzheimer’s disease (*n* = 7) patients were obtained from the Cognitive Impairment Unit, Neurology Department, University Hospital Marqués de Valdecilla (Cantabria, Spain), according to their guidelines and ethical committees’ approval. Plasma samples were obtained by centrifugation of EDTA-containing blood at 1380×*g* (5 min, 4 °C) and mixed with a protease inhibitor cocktail (Roche) before freezing at −80 °C. For human plasma samples, cognitively healthy age-matched controls, MCI, and AD-diagnosed patients were included based on clinical evaluation incorporating core AD cerebrospinal fluid biomarkers (β-amyloid 42/40 ratio, p-tau181, and total tau). No histopathological study of the brains is available for these subjects at the time the plasma sample was collected. All samples were provided after informed consent and the approval of the local ethics committee. Additional information about human samples is available in Tables S1 (hippocampal samples) and S3 (plasma samples).

### Animals

All the experimental procedures involving animals were carried out following the animal welfare guidelines of the European Union (86/609/EEC) and with the authorization of the Institutional Committee for Animal Research at the University of the Balearic Islands (CEEA-UIB) and the GOIB.

Males of the 5xFAD (Familial Alzheimer’s Disease) double transgenic mice (line Tg6799, Jackson Laboratories, USA) were used in this study. These 5xFAD mice coexpress mutant forms of human Amyloid Precursor Protein (APP) associated with Familial Alzheimer’s Disease (FAD, including the Swedish mutation (K670N, M671L), the Florida mutation (I716V), and the London mutation (V717I)) along with mutant forms of human Presenilin-1 (PS1: M146L, L286V) under the control of the neuron-specific Thy-1 promoter. The 5xFAD mice (B6/SJL genetic background) were maintained by breeding heterozygous transgenic mice with B6/SJL F1 breeders. All 5xFAD transgenic mice were heterozygous for both transgenes, and wild-type mice were used as controls.

The study analyzed paired plasma and hippocampal samples from 7-month-old WT (*n* = 5) and 5xFAD (*n* = 6). Mice were administered a lethal cocktail of ketamine/xylazine anesthesia before blood extraction by heart puncture. Blood was mixed with heparin as anticoagulant and centrifuged at 1400×*g* (5 min, 4 °C) to obtain plasma samples. After heart puncture, mice were perfused with PBS containing heparin as an anticoagulant for 10 min. Brains were immediately removed, dissected, and frozen in liquid N_2_. All samples were stored at −80°C until analysis.

### Lipid Extraction

Hippocampus pieces of ca. 100 mg (human samples) or 15 mg (mouse samples) were homogenized in a calculated volume of water using a bullet blender homogenizer (Bullet Blender Gold, Next Advance, Inc., Averill Park, NY, USA) in the presence of zirconium oxide beads (0.5 and 1 mm), at a speed level of 8 for 3 min at 4 °C to obtain a homogenate of ca. 150 mg ww/mL homogenate for human and ca. 100 mg ww/mL homogenate for mouse samples. A 10 µL portion from the hippocampus homogenate was sonicated in 750 µL (human samples) or 500 µL (mouse samples) methanol containing 7.5 µg PC (40:0) as an extraction standard and 0.001% butylated hydroxytoluene as an antioxidant in a bath sonicator for 5 min. For the plasma, a 10 µL aliquot was sonicated in 500 µL methanol containing 5 µg PC (40:0) (as extraction standard for plasma) and 0.001% butylated hydroxytoluene (as an antioxidant). Then, the mixtures were shaken for 5 min and centrifuged at 10,000 *g* for 5 min. The supernatant was transferred into a new Eppendorf tube and stored at −20 °C until MS analysis.

### Mass Spectrometry-Based Shotgun Lipidomics

Electrospray ionization (ESI)-MS analyses were performed on a high sensitivity, high-resolution Orbitrap Fusion Lumos instrument (Thermo Fisher Scientific) equipped with a TriVersa NanoMate robotic nanoflow ion source (Advion BioSciences), using chips with spraying nozzles of 5.5 µm diameter. The instrument was fully calibrated before analysis. The ion source was controlled by Chipsoft 8.3.1 software. The ionization voltages were + 1.3 kV and − 1.9 kV in positive and negative modes, respectively, and back-pressure 1 psi for both modes. The temperature of the ion transfer capillary was 260 °C. Acquisitions were performed at the mass resolution R_m/z=200_ = 240,000, and signals were recorded in centroid mode.

Based on preliminary injections, 4 µL hippocampal or 16 µL plasma extract was further diluted with 120 or 105 µL infusion solvent mixture (chloroform:methanol:iso-propanol 1:2:1, by vol.). The infusion solvent was spiked with an internal standard mix (Table S7, Avanti Polar Lipids). Next, the mixture was halved, and 5% dimethylformamide (additive for the negative ion mode) or 3 mM ammonium chloride (additive for the positive ion mode) was added to the split sample halves. Ten microliters of solution was infused and data were acquired for 2 min.

Raw MS spectra were converted to platform-independent mzML files, and lipid species were identified by LipidXplorer software [27]. Identification was made by matching the m/z values of their monoisotopic peaks to the corresponding elemental composition constraints. The mass tolerance was set to 2 ppm. Data files generated by LipidXplorer queries were further processed by self-developed Microsoft Excel macros. Quantification was made by comparing integrated signal intensities with those of the internal standards after built-in C13 isotopic corrections. Relative analyte concentrations (lipid %) were determined from the concentrations of matching internal standards (Table S7), while absolute concentrations (lipid nmol/ww mg and lipid nmol/plasma mL for hippocampus and plasma, respectively) were calculated based on the amount of the extraction standard.

Lipid species were annotated with sum formulas according to the shorthand notation for lipid structures [28]. For glycero(phospho)lipids, e.g., PC (34:1), the total numbers of carbons followed by double bonds for all chains are indicated. For sphingolipids, the sum formula like SM(36:1:2) specifies first the total number of carbons in the long-chain base and FA moiety, then the sum of double bonds in the long-chain base and the FA moiety, followed by the sum of hydroxyl groups in the long-chain base and the FA moiety. We note that PC/PE-O(x:y) (alkyl-acyl) and PC/PE-P(x:y-1) (alkenyl-acyl) species as well as PG and BMP species are isomeric. These types of isomerism were not resolved in the current work; therefore, the coexisting isomers (if any) were quantified collectively.

### Statistical Analysis

Data are expressed as the mean ± SD for graphical presentations and as mean ± SEM for the statistical summary shown in tables. For comparisons between two normally distributed groups, such as in the mouse experiments, two-tailed Student’s *t*-tests were performed. For human data involving more than two groups that were not normally distributed, the non-parametric one-way ANOVA (Kruskal–Wallis test) was performed, followed by Dunn’s post hoc multiple comparison test with Benjamin-Hochberg’s adjustment for multiple testing, as implemented in GraphPad Prism 8.0 (GraphPad Software, Inc.) software.

For volcano plot analyses, individual *p*-values from the relevant comparison were adjusted for multiple testing using the Benjamin-Hochberg procedure to control the false discovery rate (FDR). Volcano plots display log_2_ (fold change) on the x-axis and -log_10_ (adjusted *p*-value) on the y-axis, and features with FDR < 0.05 were considered statistically significant. Heatmaps were generated using Heatmapper 2 software (University of Alabama), using hierarchical clustering with Pearson distance and average linkage of the dataset.

## Results

### Human Hippocampal Lipidomic

In this study, we analyzed lipid alterations in postmortem hippocampal samples of AD patients at different Braak stages (Braak III–IV representing mild/moderate Alzheimer’s disease and Braak V–VI representing advanced AD), and age-matched Braak I–II individuals as neuropathological controls, to identify disease-associated changes. Braak stages were identified as part of the histopathological post-mortem study. The total number of lipid classes and species detected and the percentage of species showing significant alterations related to the pathological evolution for each analyzed lipid class are shown in Fig. S1. The human hippocampus sample list is shown in Table S1; the lipid classes and their relative species detected in our analysis are shown in Table S2, while the statistical significance of each lipid species is shown in Table 1.

**Table 1:** Significantly altered lipid species in the hippocampus of Braak I–II, Braak III–IV, and Braak V–VI brains

		Braak I–II			Braak III–IV					Braak V–VI				
Class	Species	Mean^a^	SEM^a^	Lipid%	Mean^1^	SEM^a^	% change	Lipid %	*P*	Mean^a^	SEM^a^	% change	Lipid %	*P*
**PC**	[36:2]	1.352	0.0937	1.54	1.089	0.0774	− 19.45	1.34	NS	1.004	0.0651	− 25.74	1.26	*
	[36:3]	0.338	0.0246	0.39	0.291	0.020	-	0.36	NS	0.267	0.0136	− 21.01	0.34	*
	[38:3]	0.136	0.0112	0.16	0.114	0.0071	-	0.14	NS	0.099	0.0053	− 27.20	0.12	**
	[38:4]	1.234	0.0438	1.41	1.002	0.0661	− 18.80	1.24	**	0.929	0.0561	− 24.72	1.17	***
	[38:5]	0.453	0.0249	0.52	0.320	0.0131	− 29.36	0.39	***	0.330	0.0124	− 27.15	0.41	***
	[40:4]	0.130	0.0061	0.14	0.110	0.0069	-	0.12	NS	0.096	0.0067	− 26.36	0.10	**
	[40:5]	0.092	0.0060	0.10	0.068	0.0026	− 26.09	0.08	**	0.065	0.0032	− 29.35	0.08	**
**LPC**	[18:1]	0.023	0.0009	0.05	0.020	0.0008	− 13.04	0.04	*	0.018	0.0011	− 21.74	0.04	*
**PE**	[34:1]	0.343	0.0123	0.37	0.296	0.0118	− 13.74	0.32	*	0.325	0.0128	-	0.35	NS
	[36:4]	0.022	0.0085	0.03	0.043	0.0053	-	0.05	NS	0.052	0.0061	136.36	0.07	**
	[38:3]	0.156	0.0105	0.18	0.118	0.0084	− 24.36	0.15	*	0.105	0.0058	− 36.69	0.13	***
	[38:4]	1.829	0.0929	2.08	1.395	0.1172	− 23.72	1.72	*	1.325	0.0872	− 27.55	1.71	**
	[40:4]	0.732	0.0389	0.83	0.597	0.0515	-	0.74	NS	0.534	0.0394	− 27.05	0.67	**
**LPE**	[22:5]	0.005	0.0009	0.01	0.003	0.0118	-	0.01	NS	0.010	0.0001	− 28.57	0.01	*
**PI**	[38.5]	0.172	0.0109	0.20	0.126	0.0073	− 26.74	0.16	**	0.136	0.0068	− 20.93	0.172	*
	[38:6]	0.012	0.0005	0.01	0.015	0.0012	-	0.02	NS	0.017	0.0009	41.67	0.021	**
	[40:6]	0.042	0.0041	0.05	0.061	0.0071	-	0.08	NS	0.070	0.0063	66.66	0.09	*
**LPI**	[18:1]	0.006	0.0004	0.01	0.004	0.0003	− 33.33	0.01	NS	0.005	0.0003	− 16.66	0.01	*
	[20:3]	0.0012	0.0001	0.001	0.0007	0.0001	− 41.66	0.001	*	0.001	0.0001	-	0.001	NS
**PG**	[38:6]	0.0013	0.0001	0.001	0.0015	0.0001	-	0.002	NS	0.0019	0.0001	46.15	0.002	*
**CL**	[68:3]	0.003	0.0003	0.003	0.002	0.0002	− 4.40	0.003	*	0.004	0.0003	-	0.01	NS
	[70:3]	0.007	0.0009	0.007	0.004	0.0005	− 46.38		**	0.005	0,0004	-		NS
	[70:4]	0.021	0.0021	0.02	0.013	0.0014	− 38.10	0.02	**	0.015	0.0009	-	0.02	NS
	[72:4]	0.029	0.0042	0.03	0.013	0.0023	− 55.17	0.02	**	0.014	0.0013	− 98.63	0.02	*
	[74:6]	0.011	0.0011	0.01	0.008	0.0007	− 27.27	0.01	*	0.008	0.0006	− 27.27	0.01	**
	[74:7]	0.040	0.0039	0.05	0.024	0.0026	− 40.00	0.03	**	0.024	0.0015	− 40.00	0.03	**
	[76:9]	0.019	0.0017	0.02	0.012	0.0013	− 36.84	0.02	*	0.013	0.0011	− 31.58	0.02	**
	[78:10]	0.002	0.0002	0.002	0.001	0.0001	− 50.00	0.001	*	0.001	0.0001	− 50.00	0.001	**
	[78:12]	0.009	0.0007	0.01	0.006	0.0007	-	0.008	NS	0.006	0.0007	− 33.33	0.01	*
	[80:14]	0.002	0.0003	0.002	0.0013	0.0002	-	0.001	NS	0.001	0.0002	− 36.84	0.001	*
**LCL**	[52:2]	0.001	0.0001	0.001	0.0003	8.2E-05	− 57.14	0.0001	*	0.0002	7.9E-05	− 71.42	0.0001	**
	[52:3]	0.002	0.0002	0.002	0.001	0.0001	− 50.00	0.001	**	0.001	0.0002	− 50.00	0.001	*
	[54:3]	0.004	0.0004	0.004	0.002	0.0002	− 50.00	0.002	***	0.002	0.0002	− 50.00	0.002	**
	[56:6]	0.003	0.0003	0.004	0.002	0.0002	− 33.33	0.002	**	0.002	0.0002	− 33.33	0.002	****
	[58:8]	0.001	0.0002	0.001	0.001	0.0001	-	0.001	NS	0.0004	0.0001	− 60.00	0.001	**
**SM**	[36:1:2]	2.868	0.1376	0.39	2.411	0.1115	− 15.93	0.42	*	2.427	0.0735	− 15.37	0.45	*
**Cer**	[36:2:2]	0.079	0.0091	0.09	0.046	0.0057	− 41.77	0.06	**	0.043	0.0034	− 45.57	0.05	**
**FFA**	[22:4]	0.069	0.0028	0.08	0.051	0.0040	− 94.20	0.06	*	0.048	0.0049	− 30.43	0.06	**
**GM1**	[36:1:2]	0.245	0.0271	0.28	0.151	0.0203	− 38.67	0.19	*	0.141	0.0132	− 42.45	0.18	**
**GD1**	[36:1:2]	5.138	0.5787	5.855	2.822	0.4121	− 45.08	3.48	*	2.730	0.3493	− 46.87	3.43	**
	[36:2:2]	0.139	0.0171	0.16	0.071	0.0141	− 48.92	0.09	*	0.073	0.0129	− 47.48	0.09	**
	[38:2:2]	0.127	0.0088	0.187	0.086	0.0117	− 32.28	0.127	*	0.109	0.0149	-	0.162	NS
	[38:2:3]	0.179	0.0251	0.20	0.073	0.0113	− 59.22	0.09	**	0.080	0.0114	− 55.31	0.10	***
	[42:2:2]	0.110	0.999	0.14	0.070	0.0078	− 36.65	0.008	*	0.095	0.0116	-	0.10	NS
**GD3**	[38:1:2]	0.021	0.0070	0.02	0.054	0.0100	157.14	0.07	NS	0.066	0.0146	214.29	0.08	*

^a^The measure of lipid species is shown as nmol/mg. Lipid%: Lipid Composition expressed as % of a particular species as compared with the whole lipidome detected. *NS* not significant, *p < 0.05 **; **p < 0.01; ***p < 0.001 as compared with the Braak I-II group. Phosphatidylcholine (PC); phosphatidylinositol (PI); lysophosphatidylcholine (LPC); lysophosphatidylethanolamine (LPE); phosphatidylethanolamine (PE); lysophosphatidylinositol (LPI); phosphatidylglycerol (PG); lysocardiolipin (LCL); cardiolipin (CL); sphingomyelin (SM); ceramide (Cer); disialoganglioside (GD1 and GD3); monosialoganglioside (GM1); free fatty acids (FFA)

**Fig. 1:**
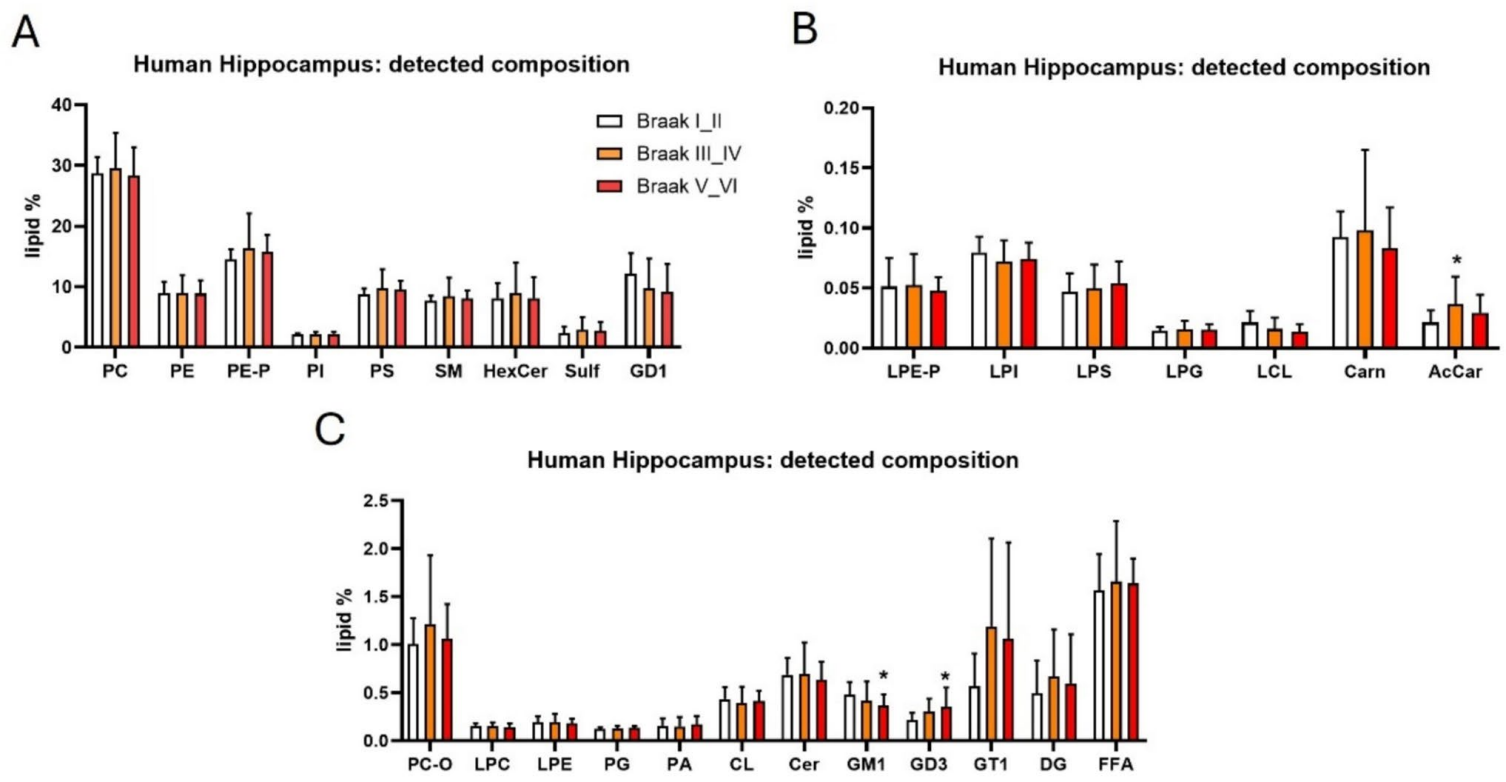
Lipid composition of human hippocampus. The bar charts represent the relative abundance of different lipid and non-lipid classes detected in human hippocampal tissue. To improve visualization, charts are split into three panels according to the relative abundance of lipid species. The y-axis shows the abundance of each class, while the x-axis lists the specific classes identified. The measure of classes is shown as lipid % as compared with the whole lipidome detected. Data are expressed as the mean ± SD from n = 11 Braak I**–**II, n = 17 Braak III**–**IV, and n = 20 Braak V**–**VI patients’ hippocampal samples. *p < 0.05; **p < 0.01; ***p < 0.001; non-parametric one-way ANOVA (Kruskal–Wallis)/Dunn’s test

The compositional analysis of the human hippocampus presented in Fig. 1 revealed a similar lipid distribution profile across different Braak stages regarding the main lipid classes. We detected only some changes in minority lipid classes, such as gangliosides. In more advanced stages (Braak V–VI), we detected a significant reduction in ganglioside GM1 class, a molecule frequently linked to neuroprotective functions [29, 30], accompanied by an increase in ganglioside GD3, which is typically associated with apoptotic signaling and neuroinflammation [31, 32] (Fig. 1). GM1 and GD3 accounted for 0.5% and 0.4%, respectively, over all lipids quantified, whereas the most abundant lipid classes such as PC (diacyl) and PE-P (PE plasmalogen) represented ca. 30% and 17% of total lipids in the sample, respectively.

Intriguingly, a detailed lipidomic analysis of the human hippocampus revealed multiple lipid species, uncovering alterations present at early/mild pathological stages (Braak III**–**IV) that persisted in later disease progression (Braak V**–**VI).

### Phosphatidylcholines

**Fig. 2:**
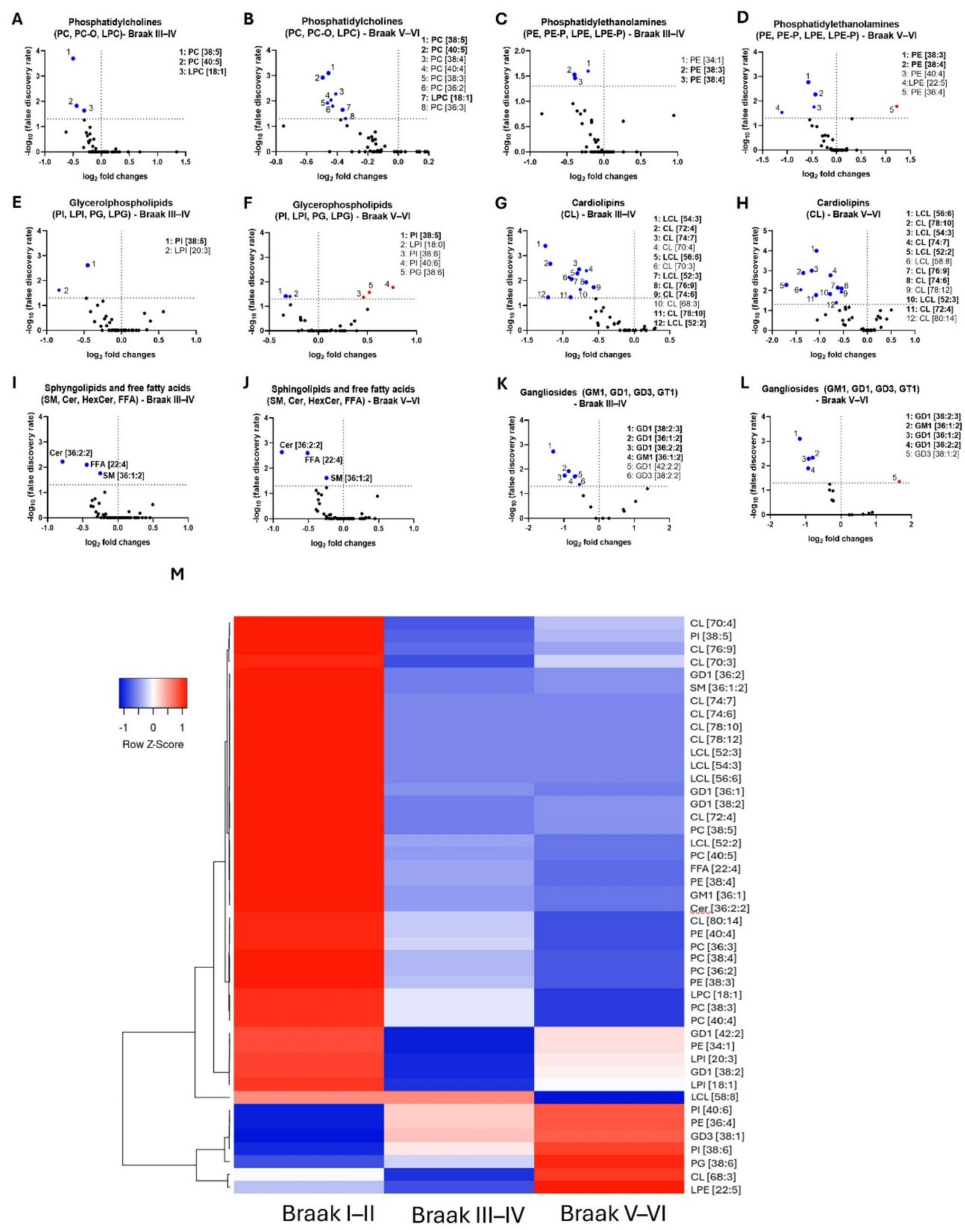
Human hippocampal lipidomics. **A**–**L** Volcano plots showing lipid species changes within lipid classes in hippocampal samples derived from Braak I–II, III–IV, and V–VI brains. Each dot on the plot represents one particular lipid species within a lipid class. Horizontal axis: fold change (in log2 scale); vertical axis: adjusted p-value (in -log10 scale). A vertical dashed line indicates a log2 fold change of 0, separating downregulated from upregulated values, while the horizontal dashed line corresponds to adjusted p-value (FDR) = 0.05. **M** Heatmap represents the lipid species altered in Braak III–IV and V–VI compared to Braak I–II. Lipids were clustered using hierarchical clustering with Pearson distance and average linkage to group lipids based on overall similarity in their profiles, facilitating visualization of patterns across samples

PC, PC-O, and LPC species were predominantly downregulated in Braak III–IV and V–VI AD patients compared to Braak I–II individuals (Fig. 2A, B, M). Our analysis revealed the downregulation of PC species [38:5] and [40:5] and LPC [18:1] in individuals at Braak III–IV (Table 1). Notably, also in Braak V–VI patients, all these species were downregulated (Table 1). Moreover, additional PC species were altered in Braak V–VI, such as PC [36:2], [36:3], [38:3], [38:4], and [40:4]. Notably, PC [36:2] and [38:4] represented the most abundant PC species (respectively, 1.54% and 1.41% of all detected lipids, Table 1).

### Phosphatidylethanolamines

Similarly, several phosphatidylethanolamine species were altered in Braak III–IV and V–VI (Fig. 2C, D, M). In particular, we observed a decreased amount of PE [34:1], [38:3], and [38:4] in Braak III–IV patients (Table 1). Similar to the alterations observed in PCs, these PE species were also significantly altered in Braak V–VI patients, and accompanied by further PE [36:4] and [40:4] and LPE [22:5] alteration (Table 1). Among them, the polyunsaturated PE [38:4] was the most abundant, showing more than 2% of total lipids detected whereas other PE family members showed abundance below 1% of total lipids (Table 1).

### Phosphatidylinositols

Phosphatidylinositol (PI) and lysophosphatidylinositol (LPI) classes showed fewer significant differences between Braak I–II, III–IV, and V–VI individuals (Fig. 2E, F, M). Specifically, PI [38:5] and LPI [20:3] were altered in Braak III–IV patients compared to Braak I–II patients (Table 1). Once more, as shown in Table 1, PI [38:5] was consistently altered in Braak V–VI patients. Additionally, in this group, we observed a downregulation of LPI [18:0] and an upregulation of PI [38:6] and [40:6].

### Phosphatidylglycerols

While no PG species showed significant changes in Braak III–IV patients, PG [38:6] was notably upregulated in the hippocampus of Braak V–VI individuals (Table 1, Fig. 2E, F, M). In this context, PG is isomeric with BMP (bis-monoacylglycerol phosphate), a marker lipid of lysosomes. Therefore, they cannot be distinguished by our shotgun lipidomic. However, based on literature data, polyunsaturated PG species such as PG [38:6] are more likely BMP species, pointing to the upregulation of these lysosomal lipid markers in AD hippocampus [33].

### Cardiolipins

Cardiolipins were within the most affected lipid species in Braak III–IV and V–VI patients when compared to Braak I–II AD patients. Curiously, cardiolipins are marker lipids of mitochondria, and mitochondrial dysfunction is widely described in AD [34, 35]. As shown in Fig. 2G, H, and M, several CLs and LCLs were found significantly decreased in Braak III–IV patients and remained affected in the Braak V–VI group. In particular, CL [72:4], [74:6], [74:7], [76:9], and [78:10] and LCL [52:2], [52:3], [54:3], and [56:6] were the species altered in both Braak groups (Table 1). Furthermore, CL [68:3], [70:3], and [70:4] were only found altered in the Braak III–IV group, while CL [78:12], [80:14], and LCL [58:8] were found altered only in Braak V–VI patients when compared to Braak I–II group (Table 1).

### Sphingolipids

These classes showed minor alterations compared to the other classes analyzed in this study (Fig. 2I, J, and M). Only SM [36:1:2] was significantly downregulated in both Braak III–IV and V–VI patients (Table 1). Of the ceramides, we were able to detect a downregulation of Cer [36:2:2], which was maintained in Braak V–VI patients (Table 1).

Gangliosides like GM1 [36:1:2], GD1 [36:1:2], [36:2:2], and [38:2:3] exhibited substantial alterations in Braak III–IV patients, which persisted into the later stages of the disease (Braak V–VI, Fig. 2K–M). GD1 [36:1:2] was the most abundant species, accounting for 5.85% of total lipids in Braak I–II and decreasing up to 3.43% in Braak V–VI stage (Table 1). Other species accounted for less than 1% of the total lipids detected in this study. Among these gangliosides, GD1 [38:2:2] and [42:2:2] were altered only in Braak III–IV patients, while GD3 [38:1:2] was altered only in the Braak V–VI group.

### Free fatty acids

No saturated or monounsaturated fatty acids were detected in our lipidomic study. Only free polyunsaturated fatty acids (PUFAs) were detected and represented around 1.5% of the total lipids detected. Among them, FFA [22:4] was downregulated in Braak III–IV when compared to the Braak I–II group. Moreover, this FFA was consistently downregulated in Braak V–VI patients (Table 1, Fig. 2I, J, M).

Together, these data reveal a progressive remodeling of lipid species composition across Braak stages in the hippocampus, characterized by a consistent downregulation of phosphatidylcholines, phosphatidylethanolamines, cardiolipins, and gangliosides—particularly pronounced in the advanced stages of Alzheimer’s disease.

### Human Plasma Lipidomic

Plasma lipidomics offers a valuable approach to uncovering systemic metabolic alterations associated with AD. Here, we compared patients with mild cognitive impairment (MCI), patients with diagnosed AD, and healthy control subjects, according to clinical diagnosis. The total number of lipid species detected (donut chart) and the percentage of species showing significant alterations related to pathology evolution for each analyzed lipid class (pie charts) are shown in Fig. S2. The human plasma sample list is shown in Table S3; the lipid classes and their relative species detected in our analysis are shown in Table S4, while the statistical significance of each lipid species is shown in Table 2.

**Table 2:** Significantly altered lipid species in the plasma of MCI, AD, and control subjects

		Control			Mild cognitive impairment					AD				
Class	Species	Mean^a^	SEM^a^	Lipid %	Mean^a^	SEM^a^	% change	Lipid%	*P*	Mean^a^	SEM^a^	% change	Lipid%	*P*
**PC-O**	[32:0]	1.45	0.101	0.013	2.18	0.248	-	0.019	NS	2.33	0.175	53.79	0.019	***
	[34:2]	7.49	0.501	0.070	8.92	1.438	-	0.077	NS	12.41	1.580	65.68	0.101	*
	[36:3]	5.34	0.409	0.050	7.07	1.138	-	0.061	NS	8.69	1.041	62.73	0.071	*
	[38:3]	0.44	0.108	0.004	0.74	0.226	-	0.006	NS	1.01	0.141	129.54	0.008	*
	[38:4]	7.80	0.424	0.073	10.97	1.192	40.64	0.095	*	12.53	1.155	60.64	0.102	*
	[40:4]	1.05	0.206	0.010	1.77	0.222	68.57	0.015	*	2.02	0.176	92.38	0.016	*
**PE**	[38:2]	5.16	0.633	0.048	6.67	0.919	-	0.058	NS	7.81	0.800	51.35	0.064	*
**PE-P**	[34:2]	1.30	0.163	0.012	1.73	0.252	-	0.015	NS	2.50	0.345	92.30	0.020	*
	[36:2]	2.96	1.374	0.028	4.07	0.458	-	0.035	NS	5.22	0.750	76.35	0.042	*
	[36:3]	1.93	0.198	0.018	2.25	0.239	-	0.019	NS	3.51	0.516	81.86	0.029	*
**SM**	[33:1:2]	5.28	0.715	0.049	8.65	1.018	-	0.075	NS	9.77	1.149	85.03	0.080	*
	[34:0:2]	4.88	0.183	0.045	5.91	0.796	-	0.051	NS	6.94	0.433	42.21	0.057	**
	[34:1:2]	157.53	4.359	1.465	204.34	17.442	29.71	1.764	*	215.04	13.495	36.50	1.751	*
	[35:1:2]	3.08	0.383	0.029	5.02	0.476	-	0.043	NS	5.65	0.726	83.44	0.046	*
	[36:1:2]	27.49	2.422	0.256	36.59	3.416	-	0.316	NS	41.52	5.465	51.03	0.338	*
	[38:1:2]	21.94	1.465	0.204	27.15	1.569	-	0.234	NS	34.08	3.745	55.33	0.277	*
	[39:1:2]	6.67	0.852	0.062	8.56	0.721	-	0.074	NS	11.94	1.580	79.01	0.097	*
	[40:1:2]	45.12	3.248	1.228	52.54	3.129	-	1.346	NS	64.49	5.649	42.92	1.424	**
	[40:2:2]	35.62	3.061	0.970	45.15	3.599	-	1.157	NS	55.37	6.080	55.44	1.22	*
	[43:1:2]	0.72	0.156	0.297	0.94	0.185	-	0.281	NS	1.39	0.212	93.05	0.332	*
**Cer**	[34:1:2]	0.48	0.035	0.004	0.81	0.130	68.75	0.007	*	0.58	0.059	-	0.005	NS
	[43:1:2]	0.61	0.042	0.006	1.07	0.225	75.40	0.009	*	1.14	0.198	86.88	0.009	*
**CE**	[16:0]	41.14	2.557	1.120	81.85	17.426	98.95	2.098	*	62.45	10.768	-	1.379	NS
**DG**	[36:3]	10.46	2.316	0.284	16.01	1.170	53.05	0.410	*	11.89	1.106	-	0.262	NS
**GM3**	[34:1:2]	2.23	0.276	0.021	3.79	0.699	69.95	0.033	*	3.33	0.359	49.26	0.027	*

^a^The measure of lipid species is shown as nmol/ml. Lipid%: Lipid Composition expressed as % of a particular species as compared with the whole lipidome detected. *NS* not significant, *p < 0.05; **p < 0.01; ***p < 0.001 as compared with the control group

**Fig. 3:**
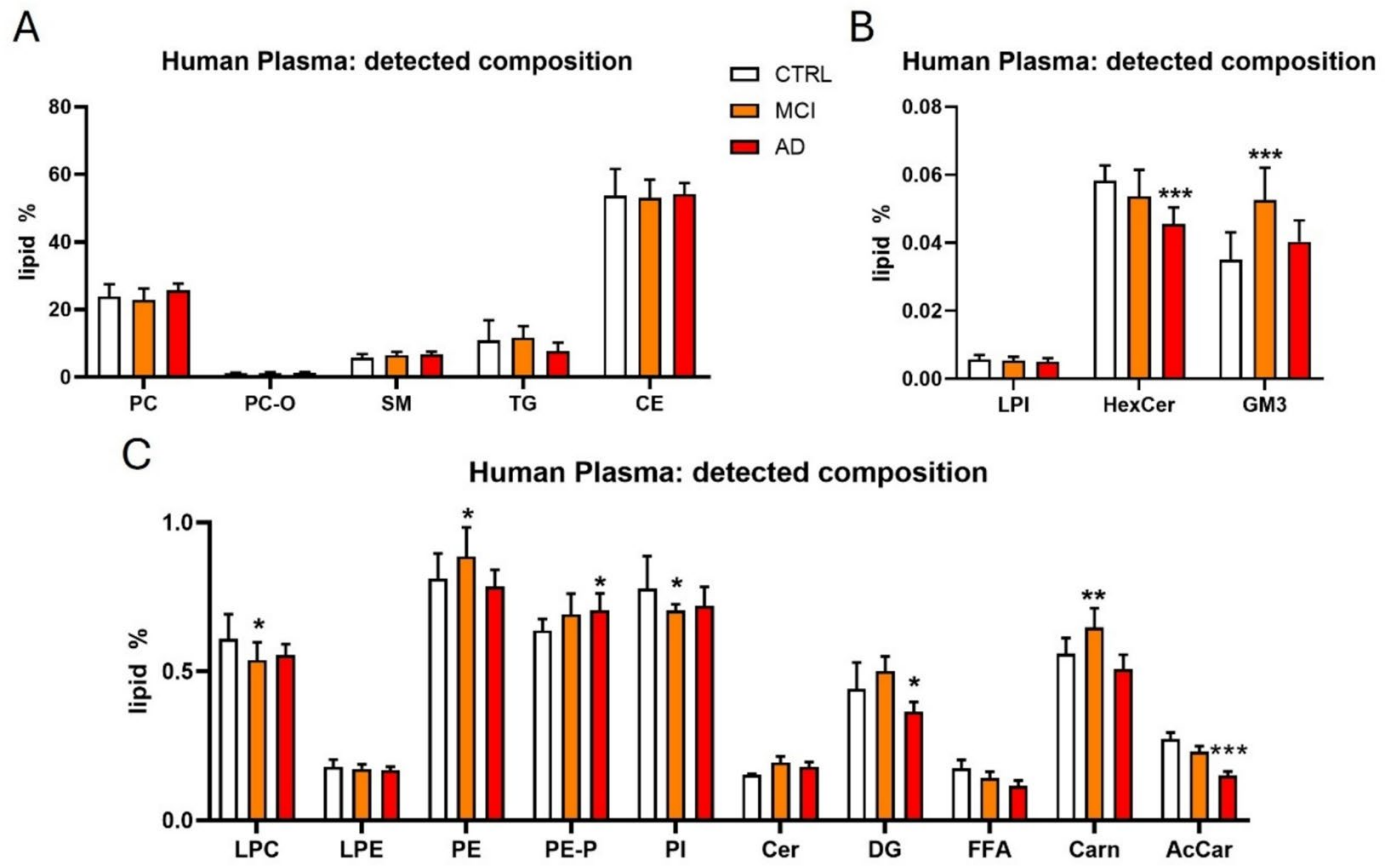
Lipid composition of human plasma. The bar charts illustrate the relative abundance of different lipid and non-lipid classes detected in human plasma. The y-axis shows the base-10 logarithm of the lipid percentage of each class, while the x-axis lists the specific classes identified. The measure of classes is shown as lipid % as compared with the whole lipidome detected. Data are expressed as the mean ± SD from *n* = 7 control (CTRL), *n* = 6 mild cognitive impairment (MCI), and *n* = 7 AD patients’ plasma samples. **p < 0.01 non-parametric one-way ANOVA (Kruskal–Wallis)/Dunn’s multiple comparison test

Lipid compositional analysis of human plasma revealed abundant lipid classes such as TG, glycerophospholipids (mainly diacyl-PC), sphingomyelin, and cholesterol esters, which are habitual components of plasmatic lipoproteins (Fig. 3). Our study also showed distinct alterations in several lipid classes across disease stages. In MCI patients, we observed changes in ganglioside GM3, frequently enriched in Alzheimer’s disease plasma (26), as well as in LPC, PE, PI, and carnitine levels (Fig. 3). In AD patients, the profile shifted toward altered levels of HexCer (glycosyl or galactosyl), PE plasmalogens (PE-P), diacylglycerols (DG), and acetylcarnitine, highlighting stage-specific lipid remodeling in plasma during disease progression (Fig. 3). Even though carnitines/acylcarnitines are not classified as lipids as such, according to their importance in lipid metabolism and as potential biomarkers [19], we included them in our analysis.

Interestingly, a more detailed lipidomic profiling of human plasma revealed a broad range of lipid species with alterations emerging in the early/mild stages of pathology and persisting as the disease progressed.

### Phosphatidylcholines

**Fig. 4:**
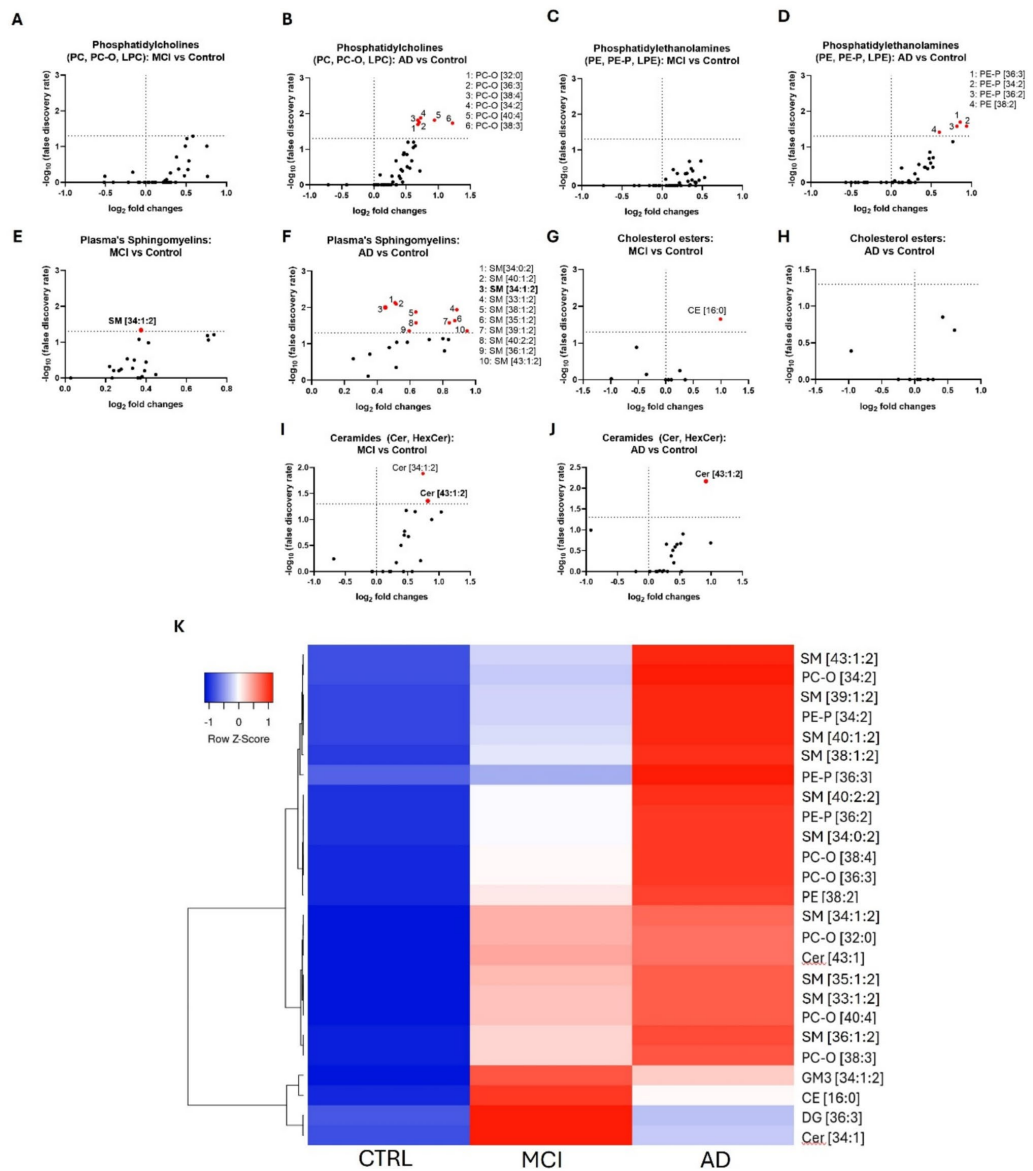
Human plasma lipidomics. **A–J** Volcano plots depict significant lipid classes in the plasma samples of MCI and AD patients compared to the control group. Each dot represents a single lipid class. The horizontal axis shows fold change (log2 scale), and the vertical axis represents adjusted p-values (-log10 scale). A vertical dashed line indicates a log2 fold change of 0, separating downregulated from upregulated values., while the horizontal dashed line corresponds to adjusted *p*-value (FDR) = 0.05. K Heatmap represents the lipid species altered in MCI and AD, versus the control samples. Lipids were clustered using hierarchical clustering with Pearson distance and average linkage to group lipids based on overall similarity in their profiles, facilitating visualization of patterns across samples. Altogether, these findings shed light on peripheral lipid dysregulation at various stages of the disease. Notably, some of the alterations detected in plasma may be connected to those occurring in the human hippocampus, pointing to potential biomarkers and pathways implicated in Alzheimer's disease progression, including phosphatidylethanolamines and phosphatidylcholines

PC (diacyl) is one of the most abundant lipid classes in plasma, accounting for about 25% of all lipids detected. However, our analysis did not detect alterations in PC species (Fig. 4A, B, K). Moreover, no LPC changes were observed within groups (Table 2). Nevertheless, PC-O species [32:0], [34:2], [36:3], [38:3], [38:4], and [40:4] were increased in AD patients when compared to the control’s subjects (Table 2).

### Phosphatidylethanolamines

Minor changes were observed within these species. In particular, three PE-P species ([34:2], [36:2], [36:3]) were found altered in AD patients but not in MCI ones (Table 2, Fig. 4C, D, K). Most of the PE (diacyl) alterations analyzed in this study were not statistically significant when compared to the control group. Although most of them showed an increasing trend, only PE [38:2] was significantly higher in AD patients. No significant differences were observed for LPE species between MCI or AD patients and control individuals Table 2.

### Sphingolipids

SM lipids exhibited the most significant changes in the plasma of MCI and AD patients, with an overall increase across all analyzed species (Fig. 4E, F, K). Specifically, SM [34:1:2] was the species we found upregulated in both MCI and AD patients. Interestingly, this species was one of the most abundant, which accounted for 1.4–1.7% of total lipids in these samples. In AD samples, several other SM species were found upregulated: SM [33:1:2], [34:0:2], [35:1:2], [36:1:2], [38:1:2], [39:1:2], [40:1:2], [40:2:2], and [43:1:2] (Table 2).

Cer tended to be more abundant in MCI than AD patients (Fig. 4I–K). In particular, Cer [34:1:2] and [43:1:2] were upregulated in MCI, while only Cer [43:1:2] maintained a significant increase in the AD group (Table 2).

Although the concentration of gangliosides in plasma is quite low, our data revealed a progressively higher abundance of the ganglioside GM3 [34:1:2] species in MCI and AD samples (Table 2). Other gangliosides showed no significant alterations related to AD progression in plasma.

### Cholesterol Esters (CE)

CE accounted for about 50% of the total amount of lipids detected in human plasma. However, we only detected an upregulation in MCI patients of CE [16:0] that was not maintained in AD group (Table 2, Fig. 4G, H, K).

### Mouse Hippocampal Lipidomic

Given the widespread use of mouse models in investigating AD mechanisms, we aimed to determine whether alterations in brain lipid composition observed in humans are similarly reflected in an AD mouse model, and whether these pathways might serve as targets for future research. It is important to acknowledge that such cross-species comparisons come with limitations due to inherent differences in species biology and disease progression. In addition, it is widely described that transgenic mouse models of AD do not replicate human pathology well, failing in several fundamental aspects [36–38].

The total number of lipid species detected (donut chart) and the percentage of them showing a significant alteration associated with pathology in 5xFAD (pie charts) are shown in Fig. S3. The lipid classes and their relative species detected in our analysis are shown in Table S5, while the concentration and statistical significance of each lipid species are shown in Table 3.

**Table 3:** Significantly altered lipid species in the hippocampus of WT and 5xFAD mice

		WT			5xFAD				
Class	Species	Mean^1^	SEM^1^	Lipid%	Mean^1^	SEM^1^	% change	Lipid %	*P* value
**PC**	[30:0]	0.10	0.0046	0.11	0.15	0.0119	53.17	0.14	**
	[32:0]	8.07	0.3707	8.93	9.17	0.2749	13.56	8.85	*
	[32:1]	0.62	0.0359	0.69	0.91	0.0247	45.55	0.88	***
	[32:2]	0.002	0.0019	0.002	0.01	0.0012	506.95	0.01	***
	[34:1]	13.05	0.5936	14.44	15.31	0.2531	17.30	14.78	***
	[34:2]	0.33	0.0182	0.37	0.51	0.0344	53.56	0.49	**
	[34:4]	0.002	0.0014	0.002	0.01	0.0014	459.68	0.01	**
	[36:2]	1.03	0.0510	1.13	1.20	0.0416	16.81	1.16	*
	[36:3]	0.18	0.0111	0.19	0.31	0.0122	72.57	0.29	****
	[36:4]	2.63	0.1274	2.90	3.08	0.0839	17.47	2.98	*
	[36:5]	0.04	0.0047	0.04	0.06	0.0033	63.74	0.06	**
	[38:3]	0.05	0.0039	0.06	0.08	0.0016	58.93	0.08	****
	[38:5]	0.83	0.0413	0.92	1.00	0.0316	20.58	0.97	**
	[38:6]	1.87	0.1015	2.07	2.19	0.0711	17.07	2.12	*
	[38:7]	0.04	0.0025	0.04	0.05	0.0012	29.95	29.95	**
	[40:5]	0.08	0.0047	0.09	0.10	0.0042	21.82	0.09	*
	[40:7]	0.37	0.0172	0.41	0.43	0.0101	14.37	0.41	*
**PC-O**	[32:0]	0.07	0.0022	0.08	0.09	0.0037	36.34	0.09	***
	[34:1]	0.18	0.0081	0.19	0.02	0.0077	27.17	0.21	**
	[36:4]	0.01	0.0013	0.02	0.03	0.0014	96.18	0.02	***
	[38:5]	0.003	0.0014	0.003	0.01	0.0017	370.17	0.02	***
	[38:6]	0.015	0.0019	0.02	0.023	0.0026	56.99	0.02	*
**LPC**	[16:0]	0.06	0.0024	0.07	0.12	0.0057	100.44	0.12	***
	[18:0]	0.03	0.0012	0.03	0.08	0.0023	158.74	0.04	****
	[18:1]	0.03	0.0006	0.03	0.04	0.0038	51.46	0.08	***
**LPE**	[16:0]	0.01	0.0008	0.01	0.02	0.0014	94.57	0.02	**
	[18:0]	0.04	0.0037	0.04	0.09	0.0081	152.59	0.09	**
	[18:1]	0.01	0.0009	0.01	0.02	0.0015	48.14	0.02	**
	[20:1]	0.0004	0.0004	0.0004	0.003	0.0007	572.76	0.003	**
	[20:4]	0.02	0.0018	0.02	0.02	0.0019	37.58	0.002	*
	[22:6]	0.03	0.0044	0.04	0.05	0.0007	46.18	0.003	*
**PE**	[32:0]	0.04	0.0026	0.04	0.05	0.0187	27.53	0.04	*
	[32:1]	0.01	0.0012	0.01	0.02	0.0015	43.65	0.02	*
	[34:2]	0.003	0.0014	0.003	0.16	0.0298	406.18	0.02	**
	[36:4]	0.26	0.0358	0.29	0.39	0.0304	48.61	0.38	*
	[38:3]	0.07	0.0086	0.07	0.97	0.0042	47.99	0.09	**
**PE-P**	[36:3]	0.08	0.0067	0.09	0.11	0.0132	35.42	0.11	*
	[38:3]	0.16	0.0151	0.18	0.21	0.0149	29.93	0.21	*
	[39:5]	0.001	0.0003	0.0006	0.003	0.0007	439.96	0.003	**
	[40:3]	0.06	0.0048	0.06	0.07	0.0029	26.39	0.07	*
**LPE-P**	[18:0]	0.01	0.0009	0.01	0.02	0.0009	56.94	0.02	**
**LPI**	[16:0]	0.004	0.0004	0.004	0.007	0.0008	108.06	0.01	**
	[18:0]	0.02	0.0030	0.02	0.003	0.0043	115.06	0.04	**
**PI**	[36:4]	0.32	0.0332	0.36	0.43	0.0251	34.63	0.42	*
	[36:5]	0.003	0.0006	0.003	0.01	0.0006	76.89	0.004	*
	[40:4]	0.01	0.0007	0.01	0.02	0.0008	11.56	0.01	**
	[40:8]	0.004	0.0005	0.004	0.01	0.0009	79.08	0.01	*
**LPS**	[18:0]	0.004	0.0004	0.004	0.01	0.0006	81.34	0.01	**
	[18:1]	0.002	0.0002	0.002	0.003	0.0002	68.87	0.002	**
	[22:6]	0.01	0.0016	0.01	0.02	0.0019	59.08	0.02	*
**PS**	[36:3]	0.007	0.0006	0.01	0.011	0.0011	51.65	0.01	*
	[36:4]	0.02	0.0014	0.02	0.02	0.0023	57.15	0.02	**
	[40:1]	0.05	0.0053	0.06	0.08	0.0067	48.92	0.07	*
	[44:10]	0.01	0.0011	0.01	0.12	0.0011	51.31	0.01	*
**LPG**	[18:1]	0.001	0.0002	0.001	0.003	0.0004	85.43	0.002	*
	[20:4]	0.0004	0.0001	0.0004	0.002	0.0001	405.65	0.001	***
	[22:6]	0.002	0.0002	0.002	0.004	0.0002	193.82	0.004	*
**PG**	[32:1]	0.001	0.00009	0.001	0.001	0.0001	52.90	0.001	*
	[34:2]	0.003	0.0002	0.003	0.004	0.0004	58.53	0.004	*
	[36:2]	0.006	0.0010	0.006	0.01	0.0001	78.23	0.01	**
	[38:5]	0.006	0.0010	0.007	0.01	0.0010	68.23	0.01	*
	[38:6]	0.003	0.0004	0.003	0.005	0.0004	49.69	0.004	**
	[40:6]	0.0004	0.0002	0.0005	0.001	0.0001	124.99	0.001	*
	[40:7]	0.004	0.0005	0.004	0.01	0.0005	95.14	0.008	***
	[40:8]	0.002	0.0003	0.002	0.005	0.0004	199.08	0.004	***
	[42:9]	0.00	0.00	0.00	0.001	0.0001	-	0.001	***
	[42:10]	0.01	0.001	0.01	0.01	0.0010	78.23	0.01	**
	[44:11]	0.00	0.00	0.00	0.001	0.0002	-	0.001	*
	[44:12]	0.03	0.0040	0.03	0.04	0.0030	44.32	0.04	*
**LCL**	[54:4]	0.0002	0.0001	0.0002	0.001	0.0001	288.77	0.001	*
**CL**	[68:2]	0.002	0.0004	0.003	0.003	0.0003	77.60	0.0003	*
	[68:4]	0.007	0.0008	0.007	0.012	0.0011	69.85	0.01	**
	[68:5]	0.001	0.0003	0.001	0.003	0.0003	188.41	0.003	**
	[70:5]	0.010	0.0011	0.010	0.016	0.0011	66.12	0.015	*
	[70:6]	0.006	0.0007	0.01	0.01	0.0010	54.78	0.01	*
	[70:7]	0.012	0.0012	0.002	0.02	0.0014	59.22	0.02	**
	[72:6]	0.015	0.0020	0.017	0.024	0.0028	57.27	0.02	*
	[72:9]	0.007	0.0005	0.01	0.011	0.0009	51.48	0.01	**
	[74:6]	0.001	0.0006	0.002	0.004	0.0007	194.29	0.004	*
	[74:11]	0.009	0.0007	0.009	0.013	0.0014	49.55	0.01	*
	[78:11]	0.001	0.0006	0.001	0.004	0.0007	276.89	0.004	*
**SM**	[34:1:2]	0.096	0.0049	0.105	0.142	0.0056	48.42	0.137	***
	[40:1:2]	0.04	0.0039	0.04	0.058	0.0040	43.67	0.06	*
**Cer**	[36:0:2]	0.003	0.0005	0.003	0.006	0.0004	80.55	0.005	**
	[36:1:2]	0.251	0.0291	0.278	0.354	0.0004	40.83	0.341	*
**GD1**	[40:2:3]	0.059	0.0289	0.165	0.126	0.0097	16.48	0.168	*
**GD3**	[36:1:2]	0.141	0.0238	0.423	0.423	0.0503	199.20	0.408	***
**GT1**	[36:1:2]	1.463	0.2561	1.618	3.00	0.2973	105.02	2.896	**
	[38:1:2]	0.359	0.0611	0.398	0.574	0.0522	59.82	0.553	*
**FFA**	[22:5]	0.003	0.0008	0.002	0.007	0.0009	154.56	0.003	*
	[22:6]	0.121	0.0081	0.134	0.169	0.0154	39.52	0.163	*
**Carn**	[0:0]	0.101	0.0054	0.111	0.007	0.0138	55.03	0.15	*

^1^The measure of lipid species is shown as nmol/mg. Lipid%: Lipid Composition expressed as % of a particular species as compared with the whole lipidome detected. NS: Not significant, *p<0.05 **; **p<0.01; ***p<0.001 as compared with the Braak I-II group

**Fig. 5:**
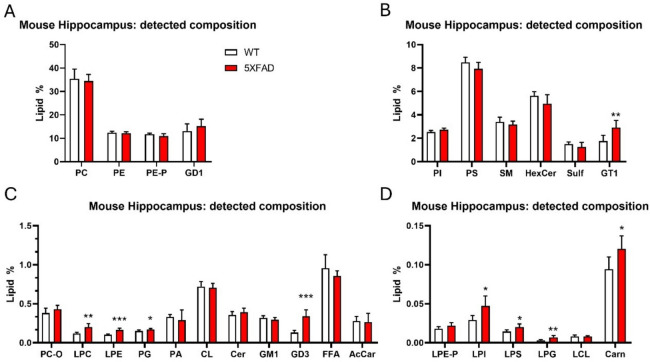
Lipid composition of mouse hippocampus. The bar charts illustrate the relative abundance of different lipid and non-lipid classes detected in mouse hippocampal tissue. To improve visualization, bar charts are split into four panels (**A**–**D**) according to the relative abundance of lipid species. The y-axis shows the abundance of each class, while the x-axis lists the specific classes identified. The measure of classes is shown as lipid % as compared with the whole lipidome detected. Data are expressed as the mean ± SD from *n* = 5 WT, and *n* = 6 5xFAD hippocampal samples. Two-tailed Student’s *t*-test: *p < 0.05, **p < 0.01, ***p < 0.001

The lipid composition (expressed as the % of total lipids detected) in WT and 5xFAD hippocampus is shown in Fig. 5. The relative distribution of lipid classes in mice and humans is highly similar. Nonetheless, mouse hippocampal samples revealed pathology-related changes in several lipid classes that were not altered in human hippocampal samples, except for GD3 that we found upregulated in the hippocampus of both AD patients and 5xFAD mice when compared to their respective control groups.

**Fig. 6:**
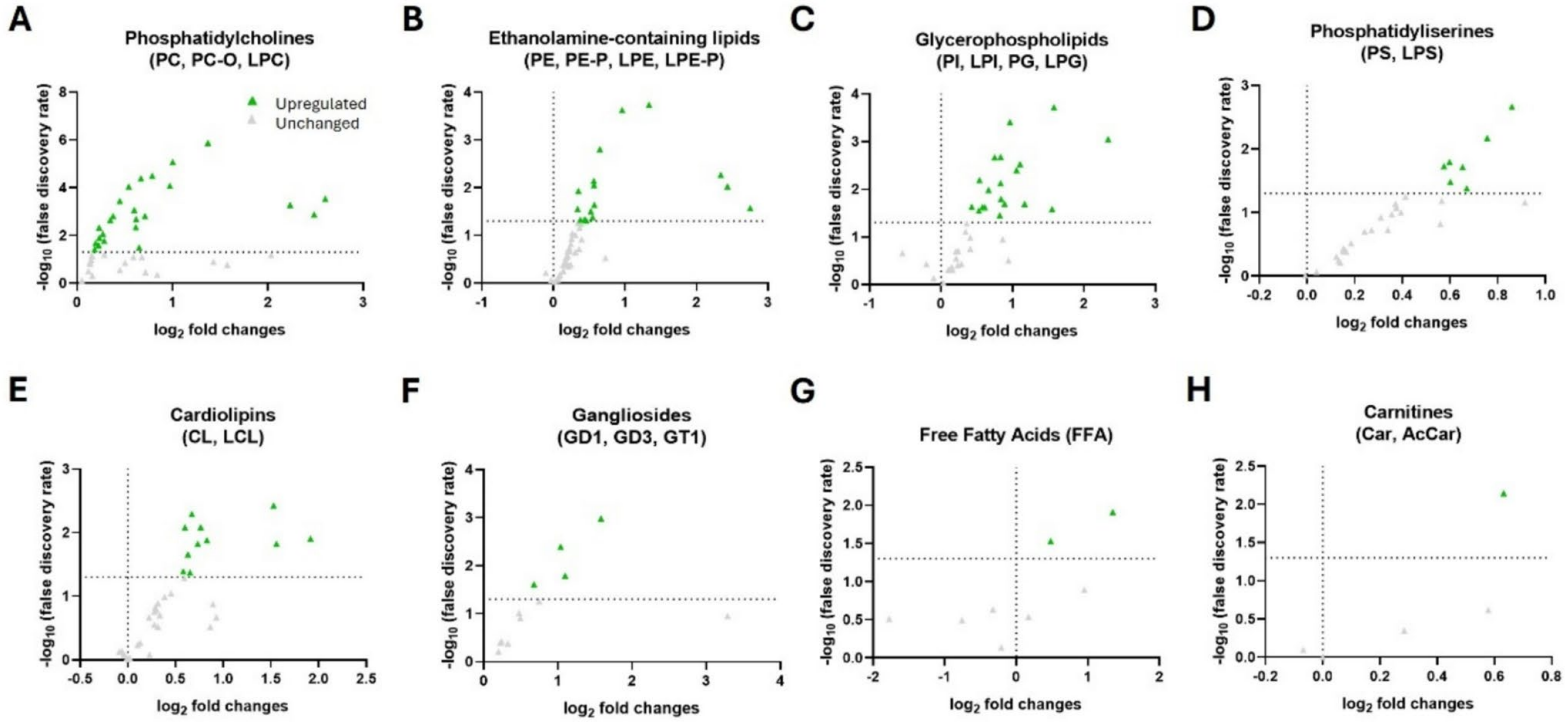
Mouse hippocampal lipidomics. **A**–**H** Volcano plots showing significant lipid species in hippocampal samples derived from WT and 5xFAD mice. Each dot on the plot is a single lipid species. Horizontal axis: fold change (in log2 scale); vertical axis: adjusted *p*-value (in -log10 scale). A vertical dashed line indicates a log2 fold change of 0, separating downregulated from upregulated values, while the horizontal dashed line corresponds to adjusted *p*-value (FDR) = 0.05

Interestingly, detailed lipidomic profiling of the mouse hippocampus revealed multiple alterations across various lipid species (Fig. 6 and Table 3). Notably, whereas the human hippocampus exhibited a pathology-related general downregulation trend in the lipid species analyzed, the mouse hippocampus showed a pronounced upregulation pattern, as previously documented [39].

### Glycerophospholipids

The analysis revealed a striking increase in phosphatidylcholine (PC)-related species in 5xFAD compared to WT mice (Fig. 6A, B). Specifically, 63.4% of diacyl-PC species, 38.5% of alkyl-PC (PC-O) species, and 60% of LPC species identified in this work were elevated in 5xFAD mice (Fig. S3 and 6A).

Phosphatidylethanolamine (PE)-containing species followed the same trend, showing a notable increase in 5xFAD hippocampus. PE and PE-P showed a moderate number of species altered (26.3% and 16%, respectively) (Fig. S3 and 6B). However, the most pronounced changes were observed in the lyso derivatives LPE and LPE-P, with 85.7% and 50% of the detected species in each class being upregulated, respectively (Fig. S3 and 6B).

Phosphatidylinositols (PIs) were also found to be increased: 26.7% of PI species and 50% of LPI species were upregulated in the 5xFAD hippocampus (Fig. S3 and 6C). Similarly, while only 15.4% of phosphatidylserine (PS) species were increased in 5xFAD mice, all analyzed LPS species (Table 3) showed increased levels (Fig. S3 and 6D).

Phosphatidylglycerol (PG) and its lyso form (LPG) were significantly altered, with 75% of LPG species and 70.5% of PG species rising in 5xFAD samples (Fig. S3 and 6C). Cardiolipins followed the uprising trend, with 20% of LCL and 32.2% of CL species increased (Fig. S3 and 6E). Similarly, SM species and ceramides were upregulated (22.2% and 20%, respectively) in 5xFAD compared to the WT mice (Fig. S3).

All altered species found in 5xFAD hippocampus are shown in Table 3 in detail. These alterations are highly discrepant with the results previously described for human AD hippocampus (compare Table 3 vs. Table 1).

### Sphingolipids

Gangliosides also shifted in 5xFAD mice, with increased numbers of GD1 (14.3%), GD3 (50%), and GT1 (66.7%) species altered (Fig. S3 and 6F). These results were partially discrepant with those obtained in human hippocampus. GD1 and GT1 species showed divergent changes between humans and mice. Whereas just one or a couple of species were upregulated in the 5xFAD samples (Table 3), respectively, several GD1 species were downregulated, and no GT1 species were affected in human samples (Table 1), as compared to the control groups. Interestingly, our data revealed that GD3 species upregulated in the 5xFAD hippocampus differ from those upregulated in human pathology, indicating species-specific variations in ganglioside alterations during disease progression between 5xFAD and human AD (compare GD3 data in Tables 1 and 3).

### Free Fatty Acids and Acylcarnitines

Free fatty acids (FFA) species were affected as well, with 22.5% of analyzed species upregulated (Fig. S3 and 6G), alongside carnitine (Fig. S3 and 6H), which also showed higher levels in the 5xFAD hippocampus.

All the altered species found in 5xFAD hippocampus are shown in Table 3 in detail. These alterations are highly discrepant with the above-described results from the human hippocampus (compare Table 3 vs. Table 1). In summary, species from the PC, PC-O, LPC, LPE, LPE-P, LPI, LPS, LPG, PG, GD3, GT1, and Carn classes significantly increased in 5xFAD hippocampus. Other lipid classes which were altered in human hippocampus such as hexosylceramides (HexCer), ganglioside GM1, acylcarnitines (AcCarn), and phosphatidic acid (PA) species remained largely unchanged between 5xFAD and WT mice (data not shown).

Taken together, the lipidomic alterations observed in the 5xFAD hippocampus substantially diverge from those found in human AD samples, with many lipid species upregulated in mice but downregulated in humans. These discrepancies likely stem from species-specific differences in lipid metabolism and neurobiology, as well as the inherent limitations of the 5xFAD model in fully replicating the complexity, progression, and cellular heterogeneity of human Alzheimer’s disease pathology.

### Plasma Lipidomic in 5xFAD Mouse Model

**Fig. 7:**
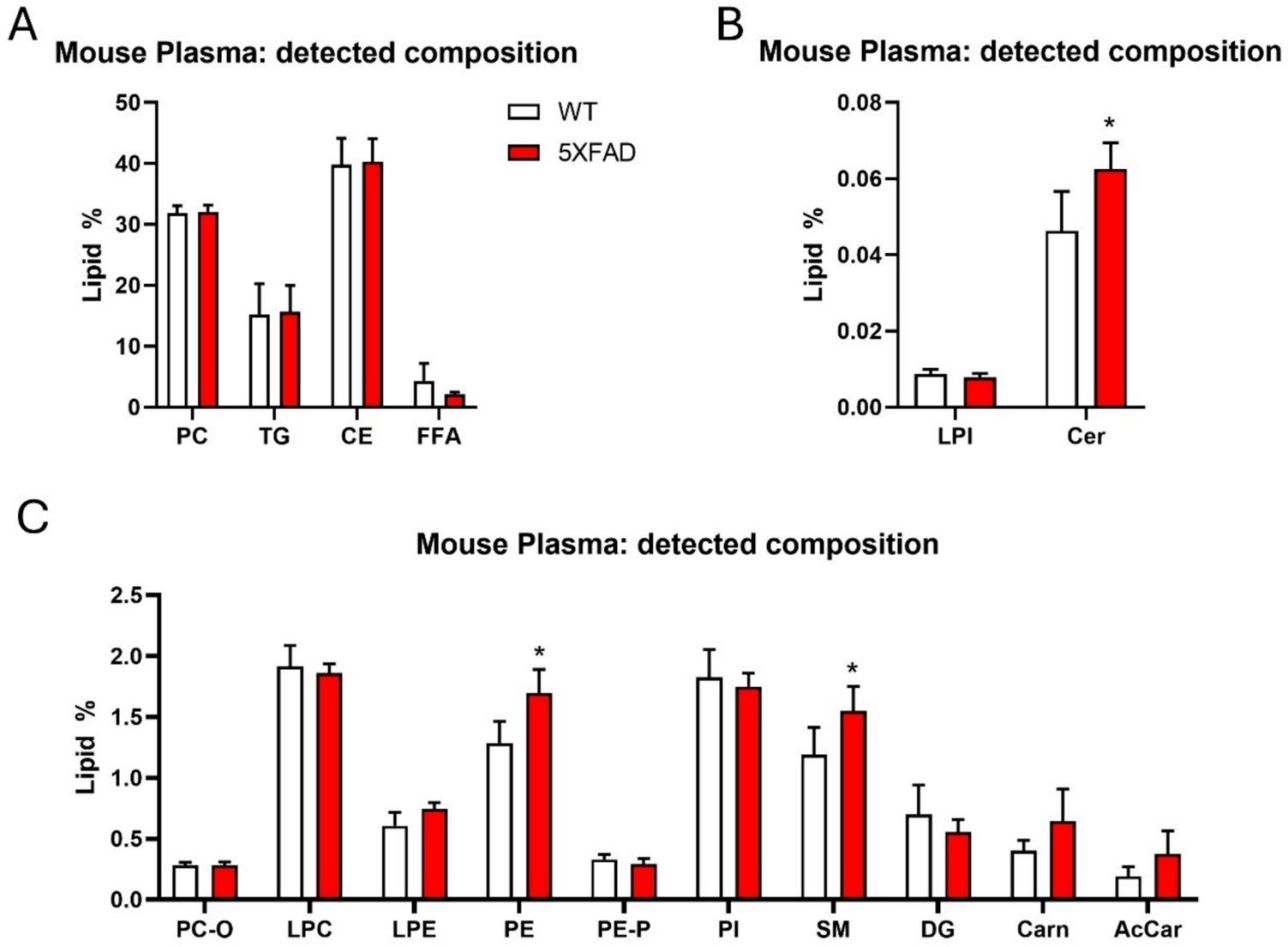
Lipid composition of mouse plasma. The bar charts illustrate the relative abundance of different lipid and non-lipid classes detected in mouse hippocampal tissue. To improve visualization, bar charts are split into four panels (**A**–**C**) according to the relative abundance of lipid species. The y-axis shows the base-10 logarithm of the lipid percentage of each class as compared with the all lipidome detected, while the x-axis lists the specific classes identified. The measure of classes is shown as lipid % as compared with the whole lipidome detected. Data are expressed as the mean ± SD from n = 5 WT, and *n* = 4 5xFAD plasma samples. Two-tailed Student’s *t*-test: *p < 0.05

After analyzing hippocampal samples, we extended our investigation to plasma samples to determine if the observed biomarkers or molecular changes were also reflected systemically (plasma composition shown in Fig. 7). The total number of lipid species detected (donut chart) and the percentage of them showing a significant alteration associated with 5xFAD pathology (pie charts) are shown in Fig. S4. The lipid classes and their relative species detected in our analysis are shown in Table S6, while the statistical significance of each lipid species is shown in Table 4.

**Table 4:** Significantly altered lipid species in the plasma of WT and 5xFAD mice

		WT			5xFAD				
Class	Species	Mean	SEM	Lipid%	Mean^1^	SEM^1^	% change	Lipid%	*P*
**PC**	[36:4]	290.01	18.322	3.329	217.23	23.989	− 25.10	3.374	*
	[38:6]	262.83	13.937	3.017	189.38	19.959	− 27.95	2.942	*
**PC-O**	[32:1]	0.57	0.040	0.007	0.38	0.042	− 33.80	0.006	*
	[34:3]	1.17	0.188	0.013	0.49	0.087	− 57.97	0.008	*
	[36:2]	1.03	0.085	0.012	0.57	0.085	− 45.28	0.009	**
	[38:5]	4.33	0.157	0.050	3.20	0.208	− 25.98	0.050	*
	[38:6]	2.28	0.157	0.026	1.68	0.208	− 26.54	0.026	*
	[40:6]	0.92	0.084	0.011	0.50	0.047	− 45.67	0.008	**
**LPC**	[20:3]	6.61	1.028	0.076	3.29	0.342	− 50.11	0.051	*
	[20:4]	17.21	2.096	0.197	10.94	0.761	− 36.41	0.170	*
	[22:6]	12.796	1.446	0.147	8.41	0.625	− 34.32	0.131	*
**PE-P**	[34:2]	1.17	0.146	0.013	0.51	0.046	− 56.64	0.008	**
	[36:1]	1.05	0.113	0.012	0.86	0.086	− 18.10	0.001	**
	[36:2]	1.10	0.117	0.013	0.16	0.094	− 85.18	0.003	**
	[36:3]	1.07	0.096	0.012	0.48	0.097	− 54.69	0.008	***
	[36:4]	2.68	0.127	0.031	1.79	0.111	− 33.41	0.028	**
	[38:4]	2.56	0.110	0.029	2.05	0.095	− 20.15	0.032	*
	[38:5]	3.01	0.225	0.034	2.19	0.215	− 27.05	0.034	*
	[38:6]	5.00	0.310	0.057	3.41	0.176	− 31.85	0.053	**
	[40:5]	3.13	0.291	0.036	2.12	0.166	− 32.28	0.033	**
	[40:6]	3.36	0.175	0.039	2.46	0.224	− 26.75	0.038	**
	[40:7]	1.08	0.085	0.012	0.64	0.101	− 41.17	0.010	**
**LPI**	[18:0]	0.10	0.011	0.0012	0.000	0.000	− 100.00	0.000	***
**PI**	[38:3]	11.33	1.618	0.130	5.31	0.520	− 53.12	0.083	*
	[38:4]	92.97	7.739	1.067	67.37	6.147	− 27.54	1.046	*
	[40:6]	1.68	0.195	0.019	0.70	0.235	− 58.15	0.011	*
**SM**	[41:1:2]	2.39	0.213	0.027	3.25	0.223	36.01	0.051	*
**Cer**	[40:2:2]	0.31	0.026	0.004	0.16	0.032	− 49.35	0.002	**
	[41:1:2]	0.23	0.031	0.003	0.38	0.052	65.94	0.006	*
	[42:3:2]	0.09	0.031	0.001	0.02	0.052	− 85.51	0.0003	*
**CE**	[20:3]	94.32	12.267	1.083	46.66	4.717	− 50.53	0.725	*
	[20:4]	1293.33	121.271	14.844	905.71	77.730	− 29.97	14.069	*
**AcCar**	[4:0]	2.16	0.165	0.025	2.71	0.135	25.05	0.042	*

^1^The measure of lipid species is shown as nmol/ml. Lipid%: Lipid Composition expressed as % of a particular species as compared with the whole lipidomedetected. NS: Not significant, *p<0.05 **; **p<0.01; ***p<0.001 as compared with the WT group

Similar to human plasma, the lipid composition of mouse plasma showed abundant lipid classes, including TG, PC (diacyl), and cholesterol esters, which are classical components of plasma lipoproteins. However, SM was less abundant in mouse plasma (ca. 1.5% vs. 6% in human plasma, Fig. 7). The compositional analysis of the 5xFAD plasma samples highlighted an increase in the concentration of Cer, PE, and SM classes. However, a detailed lipidomic analysis revealed a strong downregulation trend of several lipid species. Notably, whereas the human plasma exhibited a general upregulation trend in the lipid species analyzed, the mouse plasma showed a pronounced downregulation pattern as compared to the control groups.

### Glycerophospholipids

**Fig. 8:**
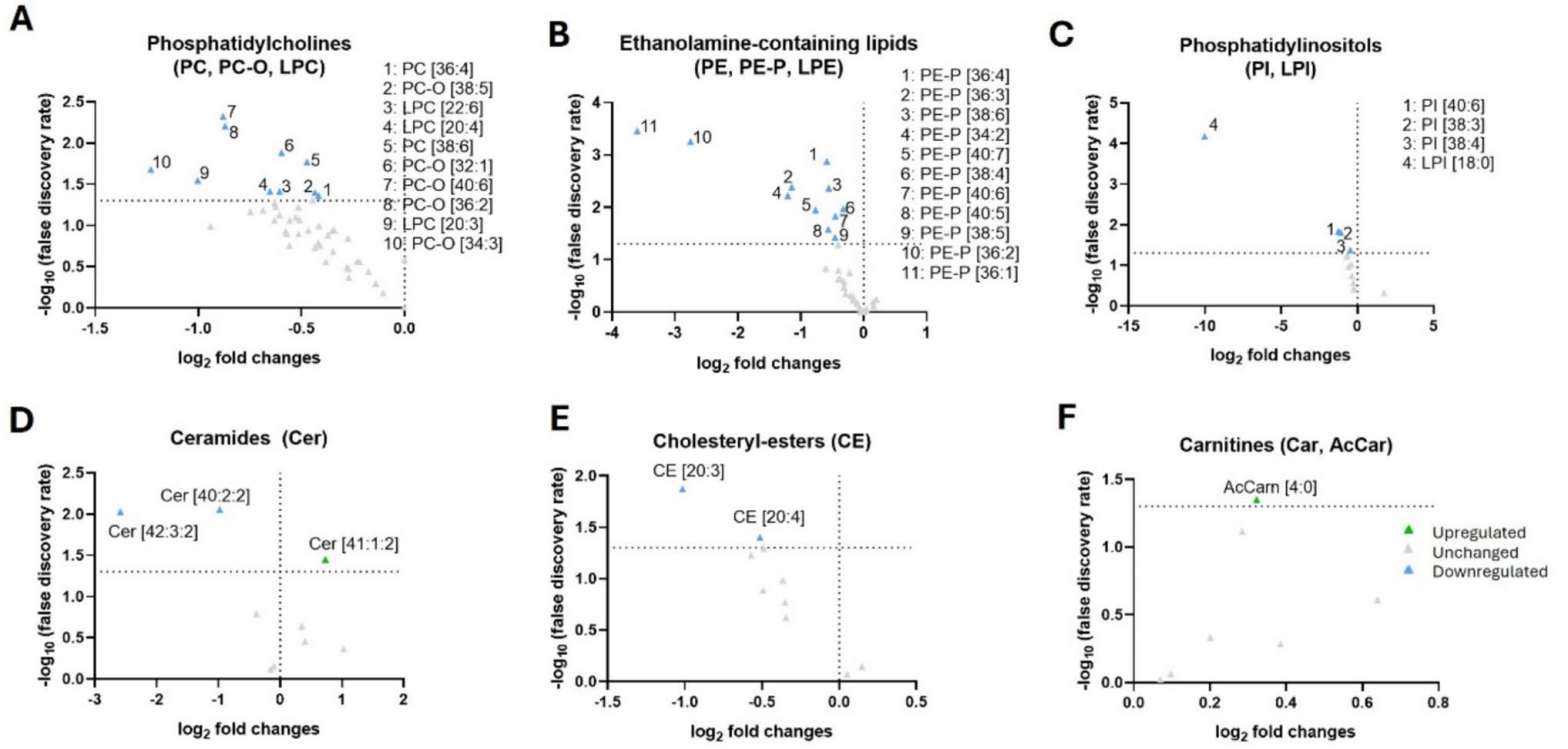
Analysis of plasma lipidomic profiles in the 5xFAD mouse model. **A**–**F** Volcano plot showing significant lipid species in 5xFAD’s plasma. Each dot on the plot is a single lipid species. Horizontal axis: fold change (in log2 scale); vertical axis: adjusted *p*-value (in -log10 scale). A vertical dashed line indicates a log2 fold change of 0, separating downregulated from upregulated values, while the horizontal dashed line corresponds to adjusted p-value (FDR) = 0.05

PC species showed limited differences, with only 7.6% of downregulated PC species (Fig. S4 and Fig. 8A). In contrast, PC-O and LPC species exhibited a broader pattern of downregulation (40% and 42.6%, respectively) of their lipid species (Fig. S4 and Fig. 8A).

LPE and PE species levels remained unchanged in 5xFAD plasma compared to WT, indicating no significant alterations in these lipid species (Table 4, Fig. 8B). On the contrary, 73.3% of the plasmalogen PE-P species exhibited a general trend of downregulation in 5xFAD plasma, with most analyzed species showing significant reductions (Fig. S4, Table 4).

PI species showed a selective downregulation, with 27.3% of PI species detected being significantly reduced in 5xFAD plasma compared to WT (Fig. S4, Table 4, Fig. 8C).

Most of these alterations described for 5xFAD plasma were discrepant with data from human plasma in AD where we showed that several glycerophospholipid species from PE and PC families remained unaltered or increased significantly according to pathology progression (Fig. S2, Table 2).

### Sphingolipids

Among the 17 species identified of SM in 5xFAD plasma, only SM [41:1:2] was found significantly upregulated (Table 4).

Among the ceramides (Cer), 33.3% of the species analyzed were downregulated in 5xFAD mice, with only Cer 41:1:2 being upregulated compared to the WT (Fig. S4, and Table 4, Fig. 8D).

### Cholesteryl Esters

In contrast to the human plasma profile, where CE levels remained relatively stable and just minor species-specific changes were observed (Tables 2 and 4), 20% of the cholesteryl esters (CE) species were significantly downregulated in the plasma of 5xFAD mice compared to wild-type controls (Fig. S4 and Fig. 8E).

### Acylcarnitines

Carnitine did not show any significant changes in 5xFAD plasma compared to WT. However, the 16.7% of acylcarnitine (AcCar) species identified was significantly upregulated (Fig. S4 and Table 4, Fig. 8F).

All altered species found in 5xFAD plasma are shown in Table 4 in detail. As for the mouse hippocampus, these alterations are widely discrepant with the results previously described for human AD plasma (compare Table 4 vs. Table 2). Nonetheless, consistent with the findings from human plasma, species from glycerolipids such as DG and TG, as well as FFAs, remained largely unchanged.

**Fig. 9:**
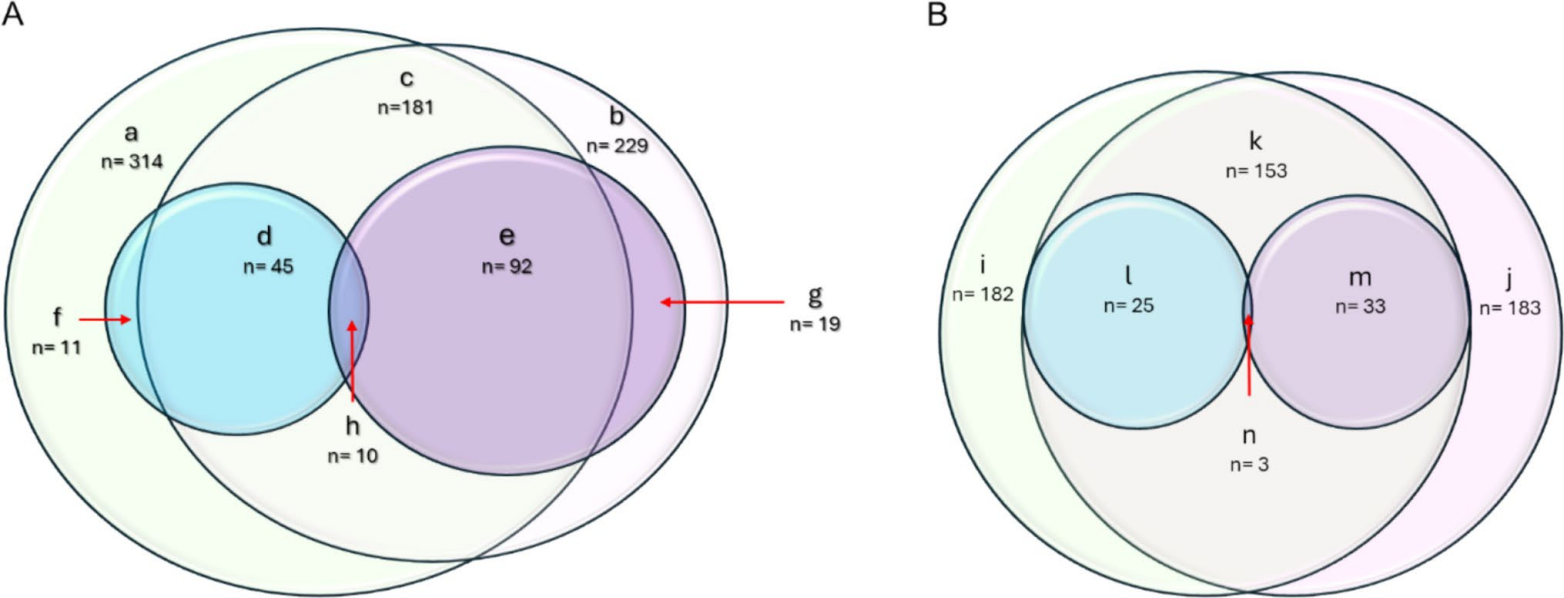
Comparative lipidomic profiles: Venn diagrams of human vs. mouse hippocampus and plasma. Lowercase letters identify each region of the diagram and indicate the number of lipid species contained in a region. **A** Overlap of lipid species detected in human and mouse hippocampus. a: lipid species detected in human; b: lipid species detected in mouse; c: lipid species detected in both human and mouse; d: lipid changes in human hippocampus; e: lipid changes in mouse hippocampus; f: lipid changes in human but not detected in mouse; g: lipid changes in mouse but not detected in human; h: lipid changes in both human and mouse hippocampus. **B** Overlap of lipid species detected in human and mouse plasma. i: lipid species detected in human; j: lipid species detected in mouse; k: lipid species detected in both human and mouse; l: lipid changes in human; m: lipid changes in mouse; n: lipid changes in both human and mouse

To visualize the overlap and specificity of lipid alterations between human and mouse datasets, we generated Venn diagrams that highlight shared and unique differentially abundant lipids across species (Fig. 9). These diagrams show that lipid species detected in a particular sample type (hippocampus or plasma) are highly conserved between humans and mice. However, the lipid species that undergo AD-related modulation differ markedly between the mouse model and human pathology, with only 10 in common in the hippocampus (PC [36:2], PC [36:3], PC [38:3], PC [38:5], PC [40:5], LPC [18:1], PE [36:4], PE [38:3], PG [38:6], CL [74:6]) and 3 in plasma (PE-P [34:2], PE-P [36:2], PE-P [36:3]) (see Fig. 9, regions marked as h and n in panels A and B, respectively). As expected, most of these species showed inverse regulation when comparing results from human and mouse samples.

Taken together, our lipidomic analysis of hippocampal and plasma samples from 5xFAD mice revealed contrasting trends: a widespread upregulation of lipid species in the brain alongside a predominant downregulation in plasma. These divergent patterns differ significantly from the lipid alterations observed in corresponding human samples, where both central and peripheral compartments often exhibit opposite trends. Nonetheless, despite these discrepancies, the lipid changes identified in both human and 5xFAD samples underscore the critical involvement of systemic and central lipid dysregulation as a hallmark of Alzheimer’s disease pathology.

## Discussion

Lipid metabolism plays a crucial role in AD, influencing key cellular processes, including membrane integrity, signaling, and energy metabolism [39]. Disruptions in lipid homeostasis have been implicated in the pathophysiology of AD, affecting neuronal function and contributing to disease progression [40, 41]. As such, lipid alterations hold promise as valuable biomarkers for early detection and potential targets for therapeutic intervention.

Given the complexity of lipid alterations in AD, it is essential to examine both plasma and hippocampal lipidomics, as these tissues offer complementary insights into disease mechanisms. Plasma lipid profiles can reflect peripheral changes, while hippocampal lipids provide a more direct view of brain-specific alterations. Furthermore, the use of both human and preclinical mouse models enhances understanding of lipid metabolism in AD and AD models, allowing for more robust comparisons and the identification of potential biomarkers and therapeutic targets.

In this study, we observed a significant downregulation of various lipid species in human hippocampal samples across Braak stages, with only a limited number of lipid species showing increased levels compared to control individuals. Noteworthy, the majority of the altered species detected in Braak stages III–IV were also persistently deregulated in Braak stages V–VI, indicating that these lipid changes may be involved in disease progression. The general decrease in lipid species in the hippocampus may be explained by several factors related to pathophysiological mechanisms of the disease [42–44]. As AD progresses, impaired cellular processes and neuronal degeneration may reduce the availability or production of several lipid species, as observed in this lipidomic analysis, leading to a decrease in their amount in the hippocampus.

The disrupted metabolism of phosphatidylcholines (PC, PC-O, and LPC) and phosphatidylethanolamine lipids (PE, PE-P, LPE) represents a key metabolic abnormality in the AD brain [45–47] [Ch. 7]. This is particularly important because, beyond their role as fundamental structural components of cell membranes [48], PC and PE serve as reservoirs for bioactive signaling molecules such as phosphatidic acid, diacylglycerol, lysophospholipids, and eicosanoid/docosanoid precursors, which are involved in various cellular functions, potentially hindering memory formation and recall. Moreover, it has been proposed that changes in PC/PE composition can influence secretase activity involved in APP cleavage, balancing toward amyloidogenic or non-amyloidogenic pathways, and this balance is strongly related to AD onset [48–50].

Likewise, polyunsaturated PCs have been associated with neuronal plasticity in neural stem cells under inflammatory stress [51] and alleviate LPS-induced systemic inflammation and cognitive impairment [52]. Accordingly, our results in human hippocampus reported a decrease of several polyunsaturated PC species that persisted until advanced stages (Braak V–VI). Some of these PC species contained five double bonds, which points towards EPA (ω−3 eicosapentaenoic acid)-containing PC species (Fig. 2).

In contrast to PC, PE is concentrated in the inner leaflet of the plasma membrane [53], and at high levels in both mitochondrial membranes [54]. The Sn-2 position of PE or PE plasmalogens is usually occupied by PUFAs such as EPA or DHA, which highlight its key role in membrane fluidity and cell signaling since the action of PLA2 releases PUFAs, which may bind to cell receptors or be converted into second messengers [55]. In agreement with our data, polyunsaturated PEs, including PE plasmalogens, have been previously reported to be significantly reduced in the brains of individuals with AD, altering membrane biophysical properties and signaling [56, 57].

Given that various neuropathological processes can contribute to a decline in PC and PE species, these reductions may be associated with the neuronal loss observed in AD. Interestingly, enhanced breakdown of PC has been previously suggested in AD [18], and increased activity of enzymes involved in the Kennedy’s pathway of synthesis has been reported in the brain of individuals with AD and Parkinson’s disease (PD), likely as a compensatory mechanism to maintain the healthy levels of these lipids [24]. Decreased levels of PC and PE species could also be caused by disrupted phospholipid trafficking or recycling, as detected by GWAS in specific genes, such as APOE, ABCA7, ABCA1, or CLU [21, 22].

We were also able to detect specific changes in PI species present in Braak III–IV and persisting in Braak V–VI patients. Certain PI, such as [36:2], [38:4], [38:5] (Table 1), and [40:5] have been reported as potential predictive markers for ischemic stroke, which is a risk factor for AD [58].

Cardiolipins (CLs) were among the most altered lipid species in the hippocampus of AD. Intriguingly, in human hippocampal samples from Braak III–IV and Braak V–VI stages, we observed a profound reduction in multiple CL species (Fig. 2; Table 1). This must be linked to disrupted mitochondrial and glial function [59, 60]. Several converging AD-related mechanisms can lead to depletion of CL species. CL is highly susceptible to peroxidation due to its polyunsaturated fatty acid content and suffers oxidative damage as a result of the increase in reactive oxygen species generated by dysfunctional mitochondria, which in turn is promoted by the direct action of the Aβ aggregates, the redox imbalance characteristic of aging, and the ApoE4 genotype, also related to AD onset [61]. Finally, the downregulation of several gangliosides, including species from the GD1, GD3, and GM1 classes, observed in this study is consistent with previous ganglioside analyses in AD brains [62].

Collectively, our hippocampal lipidomic data reveal widespread disruptions in key lipid species, highlighting profound alterations in membrane composition, mitochondrial function, and neuronal resilience that are likely to contribute to the onset and progression of AD pathology. Loss of PC/PE species and altered ganglioside composition can destabilize synaptic membranes and promote Aβ production (or seeding) at lipid rafts, while cardiolipin loss (likely due to oxidation) collapses mitochondrial bioenergetics, a central event in AD.

Interestingly, neuronal and glial membrane lipid composition and structure control the activity of proteins embedded in or interacting with membranes [17, 63, 64], thereby linking these lipid disturbances to dysfunctional cell signaling, which eventually leads to neurodegeneration and cognitive decline. On one hand, reduced PC/PE species may modify bilayer biophysical properties (thickness, curvature stress, lateral packing) at synaptic and organelle membranes [65]. Biophysical properties influence membrane proteins involved in synaptic vesicle endocytosis/recycling. Reduced PE species (important for negative membrane curvature) particularly hurt vesicle fusion/fission kinetics and might lead to reduced neurotransmitter release and synaptic failure [66]. In this context, acetylcholine (a neurotransmitter critically reduced in AD brain) can be enzymatically obtained from PC, so that the persistent decrease in several PC species could also underlie, at least in part, cholinergic synaptic dysfunction [67].

On the other hand, decreased levels of polyunsaturated PC/PE species can enhance amyloidogenic cleavage of APP, catalyzed by β- and γ-secretases in lipid rafts (membrane domains enriched with cholesterol, sphingolipids, and gangliosides) [65]. Ganglioside shifts occur with aging and in AD, and certain gangliosides (notably GM1) can inhibit Aβ oligomerization induced by sphingomyelin [68], which suggests that decreased levels of GM1 species could favor Aβ aggregation as oligomers. However, gangliosides are also well described as platforms for seeding of Aβ aggregation; thus, the effects of gangliosides on Aβ aggregation could be context-dependent [65]. Therefore, decreased levels of polyunsaturated PC/PE and GM1 species could enhance Aβ production and aggregation, key events in the onset and progression of AD, which sequentially drives tau pathology, microglia/astrocytes activation (neuroinflammation), and synaptic dysfunction leading to neuron loss according to the amyloid cascade hypothesis [50]. Finally, gangliosides such as GM1, GD1, and GD3 are essential components of neuronal membranes, involved in maintaining synaptic stability, modulating neurotrophic signaling, and protecting against oxidative stress [69, 70]. Consequently, the early decline of these gangliosides observed in hippocampal tissue may contribute to synaptic dysfunction and reduced neuronal resilience.

Within the healthy CNS, cardiolipin is a mitochondrial-specific phospholipid found almost entirely in the inner mitochondrial membrane, where it supports electron transport chain (ETC) complexes, mitochondrial dynamics, mitophagy, and regulation of apoptosis [71–73]. Cardiolipin loss initiates a vicious cycle of mitochondrial deterioration. As cardiolipin decreases, the ETC becomes less stable and less efficient, reducing mitochondrial ATP production and increasing electron leakage, which in turn enhances the generation of reactive oxygen species, leading to further cardiolipin oxidation, deepening structural and functional damage to mitochondria, and intensifying bioenergetic failure that contributes to neuronal degeneration [74, 75].

Along with findings in the hippocampus, plasma samples from MCI and AD patients showed a pronounced increase in several lipid classes and species compared with those from control subjects. This suggests a systemic lipid imbalance emerging as early as the MCI stage, which progressively intensifies in AD patients, potentially contributing to or reflecting the ongoing neurodegenerative processes. The increase in specific lipids observed in plasma could be linked to a response from peripheral tissues. As an example, lipid metabolism in peripheral organs such as the liver has been shown to be altered in response to neurodegeneration, potentially increasing plasma levels of specific lipids [76–78]. Thus, as the brain becomes less efficient at maintaining its lipid balance, liver lipid metabolism may shift, leading to an accumulation of specific lipids in the bloodstream. This systemic response is likely mediated by chronic peripheral inflammation related to AD progression, characterized by elevated circulating levels of pro-inflammatory mediators [79], which stimulate the liver to increase lipogenesis and enhance the secretion of lipoproteins via upregulation of SREBP-1c, inhibition of insulin signaling, and increased expression of ApoB [77]. This output of lipoprotein from the liver may further disrupt plasma lipid composition and interfere with the supply of structural lipids (phospholipids, plasmalogens, essential fatty acids) from the periphery to the brain through blood–brain barrier disruption, thus contributing to the cerebral lipid alterations, which are related to synaptic dysfunction, mitochondrial impairment, and compromised neuronal resilience [80]. In summary, this contrast between hippocampal and plasma lipid levels highlights the complex interplay between central and peripheral lipid metabolism in AD.

The most pronounced changes in plasma involved SMs and PC-O species (Table 2). Higher plasmatic levels of SM species were associated with an increased risk of AD among men but a reduced risk of AD among women according to the longitudinal Baltimore Study [81]. Moreover, alterations in plasma PC-O levels have been linked to both AD pathology and disease progression [82], supporting the relevance of our data. The persistence and amplification of these changes from MCI to AD suggest a progressive shift in lipid homeostasis that may correlate with disease severity. In addition to SM and PC-O, other lipid species from PE, PE-P, CE, and ceramides exhibited a significant upward trend. The increase in certain ceramide species is particularly relevant, as these lipids are known mediators of apoptosis [83, 84], neuroinflammation [85, 86], and synaptic dysfunction [87], all of which contribute to AD progression. Notably, previous studies analyzing the molecular profiles of the ceramidome revealed a significant increase in specific Cer species in the plasma of AD patients [13, 87], which is consistent with our results. Together, these findings emphasize the need for a multi-faceted approach. Integrating both central and peripheral lipid profiles can help elucidate the mechanisms underlying AD, identify potential therapeutic targets, and discover peripheral biomarkers for early detection and disease monitoring.

In 5xFAD mice, we detected significant lipid alterations in both the hippocampus and plasma. However, these changes were widely uncorrelated with those observed in human patients with mild cognitive impairment (MCI) and AD. Our data revealed opposing trends, with elevated levels of many lipid species in the hippocampus and reduced levels in plasma as compared to healthy animals. Only a minimal number of lipid species were regulated in the same direction in both human AD and 5xFAD samples. Despite these discrepancies, lipid dysregulation remains a shared hallmark of AD, and understanding these variations is therefore essential for correctly interpreting findings from preclinical models and assessing their relevance to human disease.

These discrepancies may be explained in part because of fundamental interspecies differences in lipid metabolism. In this sense, although our results showed a clear parallelism between lipid profiles in human and mouse (hippocampus or plasma), particular differences between them were also evident. Indeed, not all lipid species detected in mice were found in human samples (Fig. 9). On the other hand, observed lipid variations can be attributed to differences between human pathology and the pseudopathology of humanized models (usually genetically modified models) of the disease. 5xFAD is a PS1/APP model based on the overexpression of mutated forms of human APP and PS1. This model was designed to induce a human amyloid pathology in the mouse brain. Therefore, all lipid changes observed in 5xFAD mice can be attributed to the inserted transgenes, amyloid pathology, or the consequences derived from both. In this sense, in the present study, we detected increased levels of a variety of lipid species in 5xFAD hippocampus (Table 3): widespread alterations in phospholipids, sphingolipids, carnitine, and several ganglioside species that highlight probable disruptions in membrane homeostasis, lipid signaling, neuroinflammation, and energy metabolism [88]. In contrast to 5xFAD, AD in humans is mainly sporadic (not related to FAD clinical mutations), and the causes leading to pathology are still largely unknown. In this context, lipid alterations in AD may be part of the multifactorial etiology of the disease, and they are likely involved in the onset of the disease [89]. Therefore, lipid alterations in human pathology and humanized models may have different origins.

Discrepancies between Alzheimer’s pathology and humanized models of AD have been widely described, and these models show many limitations in replicating the human pathology [90]. Neurodegeneration is minimal or absent in many transgenic models, unlike the extensive neuronal loss described in patients [91, 92]. Furthermore, synaptic loss and plasticity deficits in patients are only partially reproduced in these animals [93–95]. Consequently, the different lipid dysregulation observed between 5xFAD and human disease is an additional discrepancy, consistent with broader evidence of lipid dysregulation observed in transgenic AD models and reported by other authors [96–98]. However, lipid dysregulation in these models has been related to enhanced amyloid pathology [65], synaptic dysfunction [99], neuroinflammation [100], and oxidative stress [101], all of which are central to AD. Together, these findings underscore the heterogeneity among AD mouse models and highlight the need for model-specific interpretations when investigating lipid alterations and their relevance to human disease.

In contrast to 5xFAD hippocampus, we detected a general downregulation of several lipid species in 5xFAD plasma samples, including phospholipids, ceramides, and carnitine species (Table 4). This systemic lipid deficiency may reflect increased lipid utilization or impaired lipid mobilization, potentially linked to early metabolic dysfunction and peripheral inflammatory responses in AD [90, 98, 102]. Reduced phospholipid and ceramide levels in plasma highlight altered lipid transport mechanisms, disrupted lipoprotein metabolism, or compensatory redistribution of lipids between the brain and peripheral tissues [98, 103–105]. Given the critical role of these lipids in cellular homeostasis, their dysregulation in circulation may contribute to systemic metabolic alterations associated with neurodegenerative processes.

The results observed in 5xFAD models underscore the complexity of lipid metabolism in AD, with opposing trends observed in the brain and peripheral circulation. The species-specific accumulation of lipids (particularly phosphatidylcholines, phosphatidylethanolamines, phosphatidylglycerols, cardiolipins, and gangliosides) in the 5xFAD hippocampus aligns with the known role of lipid dysregulation in Alzheimer’s disease, contributing to altered membrane composition, neuronal, glial, and microglial dysfunction, and blood–brain barrier disruption [64, 105–108]. Thus, these species-specific accumulations in the 5xFAD hippocampus might represent a very early stage of AD [109, 110].

Importantly, these alterations represent promising therapeutic targets, as interventions aimed at restoring lipid balance may reverse the altered biophysical properties of neuronal membranes in AD [64].

## Conclusions

Direct lipidomic comparisons of human plasma and hippocampus are absent in the current literature. Most studies analyze either plasma or brain or compare animal brain to human brain. To our knowledge, this is the first study to perform a parallel lipidomic analysis of human plasma and hippocampal samples across AD progression, offering novel insights into the relationship between central and peripheral lipid alterations in AD.

Our analysis highlights novel candidate lipid biomarkers to be used in clinical settings, such as specific plasma alterations in several PC-O and SM species, that hold significant potential for the early detection of AD. The presence of these lipid alterations as early as the MCI stage suggests that disruptions in lipid metabolism arise early in the disease process, during the prodromal phase of AD, underscoring the potential of plasma lipidomic as a powerful tool for identifying early peripheral biomarkers before significant cognitive decline occurs. Moreover, the altered lipid classes and species identified in both hippocampal and peripheral tissues in our study align with growing evidence that lipid dysregulation is a key contributor to AD pathogenesis, extending beyond the conventional amyloid and tau-focused hypothesis. These lipid alterations present promising opportunities for therapeutic intervention targeting lipid metabolism and membrane dynamics. Lipid-based therapies could lead to novel approaches for preserving neuronal function and improving patient outcomes.

## Limitations

While our study provides valuable insights into lipid alterations associated with Alzheimer’s disease progression, there are limitations to consider. First, although we identified promising biomarkers, the relatively small sample size in both human (53 hippocampal samples, 20 plasma samples) and mouse cohorts (11 individuals) limits the generalizability of our findings. However, the data provides a strong foundation for exploring a broader range of individual lipids in future studies, paving the way for deeper insights into lipid alterations in AD.

Human brain samples classified as Braak I–II are being used as neuropathological controls in the present work. Although the probability of AD is very low in these samples, this does not completely rule out the possibility of cognitive impairment; however, such cognitive impairment should not be caused by AD. In addition, the possible presence of other pathologies could also bias data interpretation from human brain and plasma samples. Therefore, some caution must be taken when interpreting data from these samples.

Our study provides insights into exploring clinical biomarkers for AD, as plasma lipids are relatively easy to extract. However, changes in plasma lipid levels may also reflect damage or pathology in peripheral organs, which can limit their direct association with brain pathology. To address this limitation, we integrated lipidomic analyses of both hippocampal tissue and plasma from AD patients across different pathological stages. This combined approach enables the identification of lipid alterations shared between central and peripheral compartments, improving our understanding of disease progression and supporting potential biomarker discovery. Further independent validation in cohorts with pathology-confirmed diagnoses is needed.

Finally, while our lipidomic approach identified significant alterations, the functional implications of these lipid changes on brain pathology and cognitive decline remain to be fully elucidated. Future studies should aim to investigate the mechanistic implications of these lipid alterations, particularly their impact on synaptic integrity, mitochondrial function, and neuroinflammation.

## Supplementary Information

Below is the link to the electronic supplementary material.

ESM 1 (649 KB JPG)

ESM 2 (1.18 KB JPG)

ESM 3 (1.06 KB JPG)

ESM 4 (368 KB JPG)

ESM 5 (15.5 KB DOCX)

ESM 6 (23.6 KB DOCX)

ESM 7 (29.7 KB DOCX)

ESM 8 (16.7 KB DOCX)

ESM 9 (15.9 KB DOCX)

ESM 10 (16.5 KB DOCX)

ESM 11 (15.5 KB DOCX)

ESM 12 (23.2 KB DOCX)

## Data Availability

All data generated or analyzed during this study are included in this article and its supplemental data.

## Abbreviations

AD: Alzheimer’s disease
MCI: Mild cognitive impairment
CNS: Central nervous system
PC: Phosphatidylcholine
PC-O: Ether-linked phosphatidylcholines
PI: Phosphatidylinositol
PE-P: Ether-linked phosphatidylethanolamine
LPC: Lysophosphatidylcholine
LPE: Lysophosphatidylethanolamine
LPE-P: LPE with alkenyl substituents
PE: Phosphatidylethanolamine
LPI: Lysophosphatidylinositol
LPS: Lysophosphatidylserine
PS: Phosphatidylserine
LPG: Lysophosphatidylglycerol
PG: Phosphatidylglycerol
PA: Phosphatidic acid
LCL: Lysocardiolipin
CL: Cardiolipin
SM: Sphingomyelin
Cer: Ceramide
HexCer: Hexosylceramide: glycosyl- or galactosylceramides
Sulf: Sulfatides
GD1 and GD3: Disialoganglioside
GM1: Monosialoganglioside
GT1: Trisialoganglioside
FFA: Free fatty acids
Carn: Carnitine
AcCar: Acylcarnitine
CE: Cholesteryl ester
DG: Diglyceride
TG: Triglyceride
