# Supplementary figures and images for: Lipidomic Analysis of Human Plasma and Hippocampus Across Alzheimer’s Progression and Preclinical 5xFAD Mouse Model
Source: Mol Neurobiol. 2026 Apr 13;63(1):561. doi: 10.1007/s12035-026-05849-1 (PMC13076374; doi:10.1007/s12035-026-05849-1)

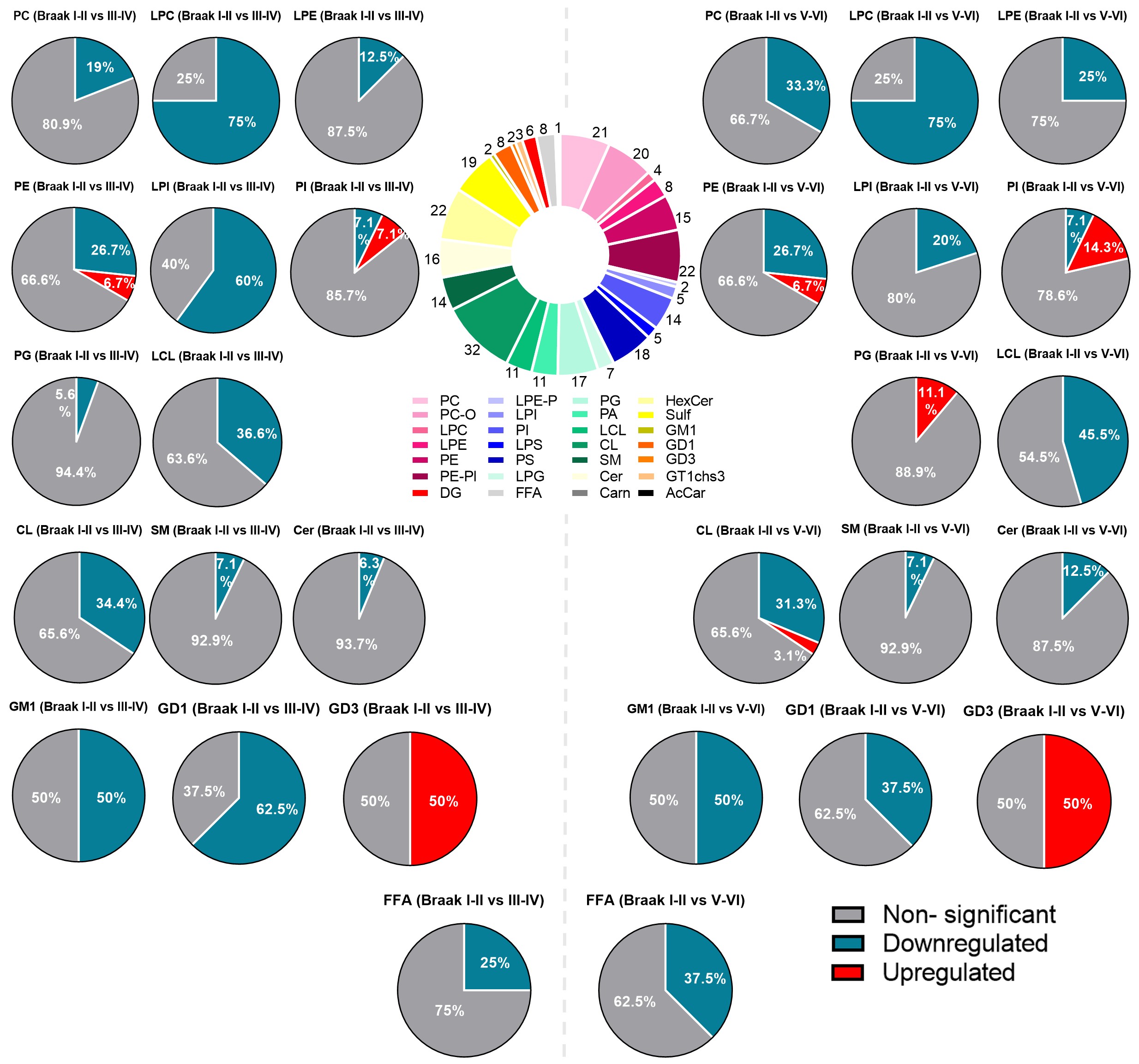

Supplement: Supplementary file 1 — (649 KB JPG) [file 12035_2026_5849_MOESM1_ESM.jpg]

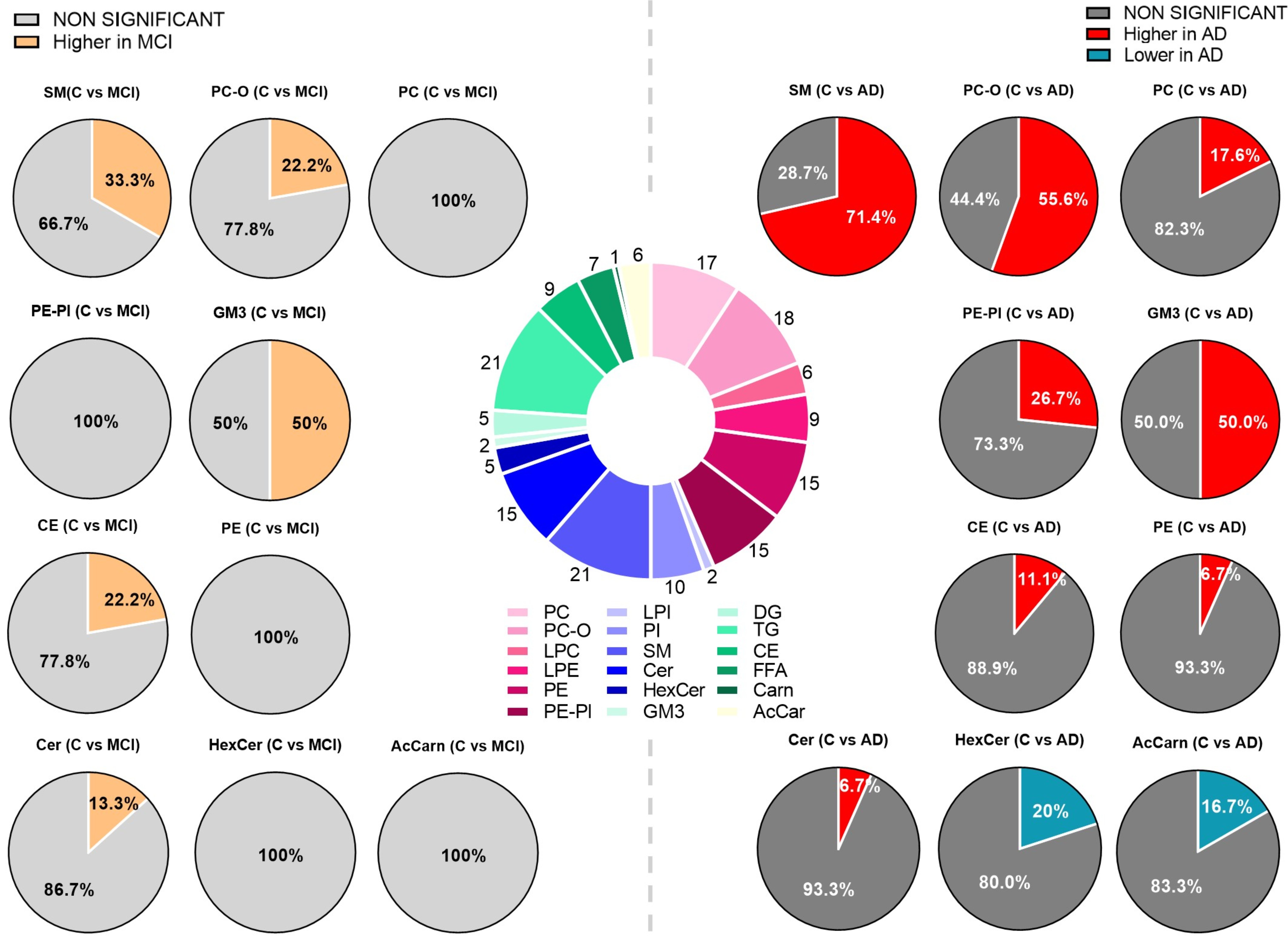

Supplement: Supplementary file 2 — (1.18 KB JPG) [file 12035_2026_5849_MOESM2_ESM.jpg]

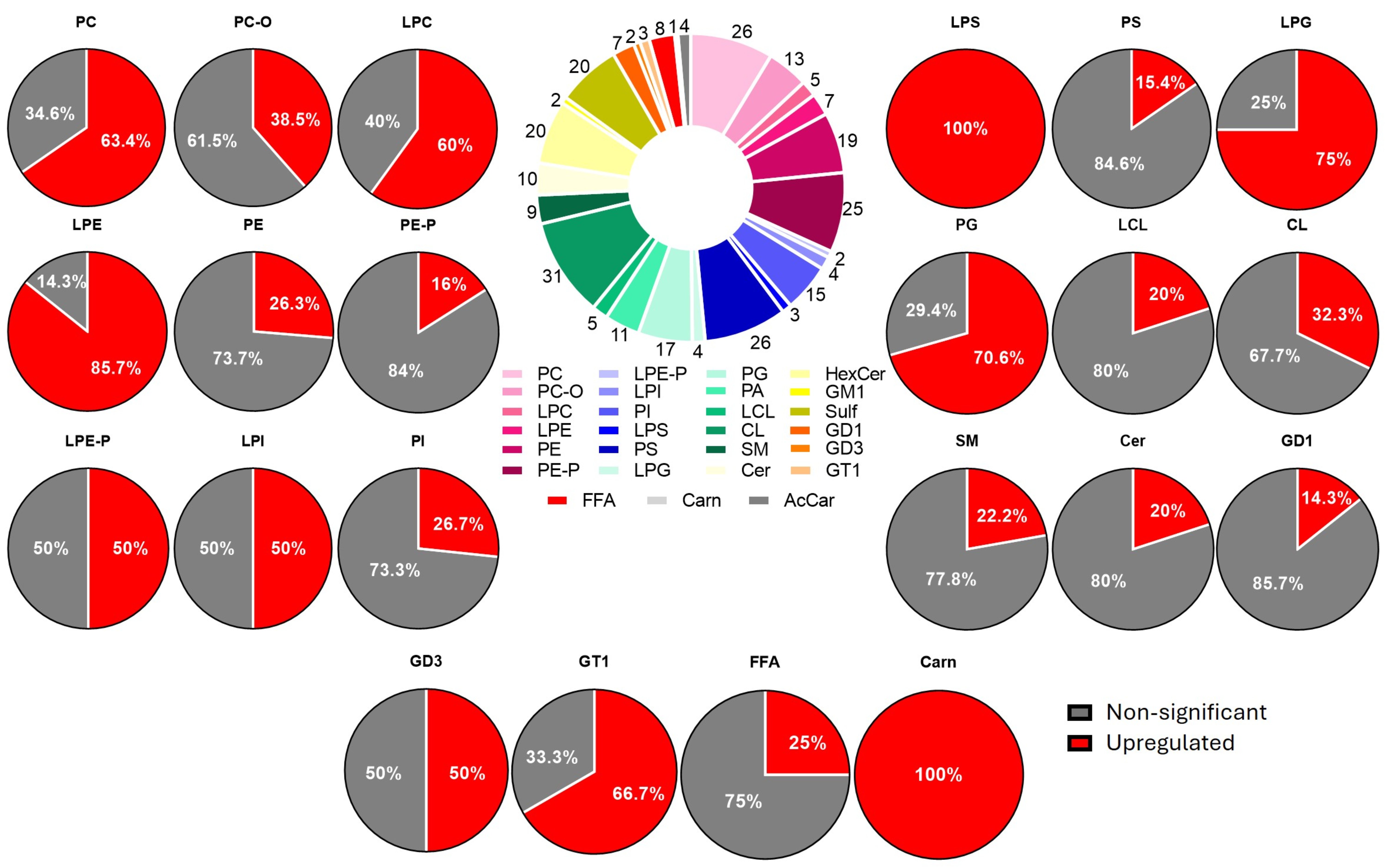

Supplement: Supplementary file 3 — (1.06 KB JPG) [file 12035_2026_5849_MOESM3_ESM.jpg]

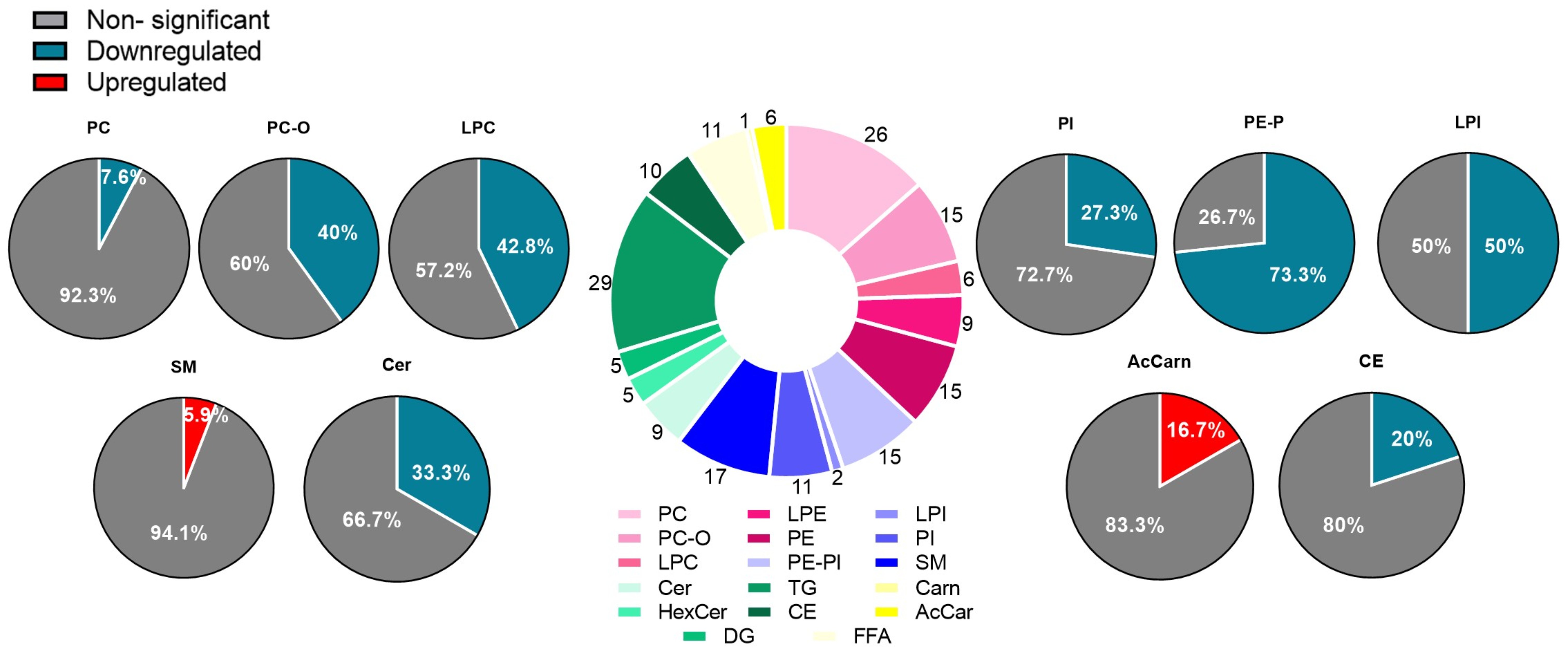

Supplement: Supplementary file 4 — (368 KB JPG) [file 12035_2026_5849_MOESM4_ESM.jpg]
