# Supplementary material for: Lipidomic Analysis of Human Plasma and Hippocampus Across Alzheimer’s Progression and Preclinical 5xFAD Mouse Model
Source: Mol Neurobiol. 2026 Apr 13;63(1):561. doi: 10.1007/s12035-026-05849-1 (PMC13076374; doi:10.1007/s12035-026-05849-1)
Supplement: Supplementary file 5 — (15.5 KB DOCX) [file 12035_2026_5849_MOESM5_ESM.docx]

Fig.S1. Lipids in the human hippocampus. The central donut chart illustrates the total number of lipids analyzed across different lipid classes in human Hippocampus (classes represented by different colours). Numbers around the donut chart represent the amount of lipid species analyzed. The accompanying pie charts represent the percentage distribution of various lipid classes across hippocampal samples of Braak III-IV (right panel), and Braak V-VI patients (left panel), compared to the control group (Braak I-II patients). Phosphatidylcholine (PC); Ether-linked Phosphatidylcholines (PC-O); Phosphatidylinositol (PI); Ether-linked Phosphatidylethanolamine (PE-P); Lysophosphatidylcholine (LPC); Lysophosphatidylethanolamine (LPE); LPE with alkenyl substituents (LPE-P); Phosphatidylethanolamine (PE); Lysophosphatidylinositol (LPI); Lysophosphatidylserine (LPS); Phosphatidylserine (PS); Lysophosphatidylglycerol (LPG); Phosphatidylglycerol (PG); Phosphatidic acid (PA); Lysocardiolipin (LCL); Cardiolipin (CL); Sphingomyelin (SM); Ceramide (Cer); Hexosylceramide (HexCer): glycosyl- or galactosyl- ceramides; Sulfatides (Sulf); Disialoganglioside (GD1 and GD3); Monosialoganglioside (GM1); trisialoganglioside (GT1); Free Fatty Acids (FFA); Carnitine (Carn); Acylcarnitine (AcCar); Cholesteryl ester (CE); Diglyceride (DG).

**Fig. S2. Lipids in human plasma.** Central donut chart shows the total number of lipids analyzed across different lipid classes in human plasma. Numbers around the donut chart represent the amount of lipid species analyzed. The accompanying pie charts present the percentage distribution of various lipid classes across plasma samples of Mild Cognitive Impairment (MCI, left charts) patient, and Alzheimer's Disease (AD, right chars) patient compared to the control group.

Fig. S3. Lipids in mouse hippocampus. Central donut chart shows the total number of species detected in each lipid class from mouse hippocampal samples. Numbers around the donut chart represent the amount of lipid species analyzed. The accompanying pie charts present the percentage distribution of various lipid classes across hippocampal samples of 5xFAD compared to the control group.

Fig.S4. Mouse plasma lipidomics. Central donut chart illustrates the total number of lipids analyzed across different lipid classes in mouse plasma. Numbers around the donut chart represent the amount of lipid species analyzed. The accompanying pie charts present the percentage distribution of various lipid classes across plasma samples of 5xFAD compared to the control group.
