# Supplementary material for: Lipidomic Analysis of Human Plasma and Hippocampus Across Alzheimer’s Progression and Preclinical 5xFAD Mouse Model
Source: Mol Neurobiol. 2026 Apr 13;63(1):561. doi: 10.1007/s12035-026-05849-1 (PMC13076374; doi:10.1007/s12035-026-05849-1)
Supplement: Supplementary file 6 — (23.6 KB DOCX) [file 12035_2026_5849_MOESM6_ESM.docx]

| **Table S1. Human hippocampus sample list** | | | | | | | | |
| --- | --- | --- | --- | --- | --- | --- | --- | --- |
| ID NUMBER |  | A/P DIAGNOSIS |  | AGE |  | SEX |  | PM DELAY (hours:min) |
| A10/098 |  | AD BRAAK STAGE I |  | 73 |  | FEMALE |  | 17:45 |
| A11/102 |  | AD BRAAK STAGE I |  | 66 |  | MALE |  | 12:10 |
| A11/075 |  | AD BRAAK STAGE I |  | 61 |  | MALE |  | 04:30 |
| A11/084 |  | AD BRAAK STAGE I |  | 77 |  | MALE |  | 06:55 |
| CS1694 |  | AD BRAAK STAGE II |  | 58 |  | MALE |  | 05:00 |
| CS1468 |  | AD BRAAK STAGE II |  | 64 |  | MALE |  | 10:00 |
| CS1858 |  | AD BRAAK STAGE II |  | 83 |  | FEMALE |  | 07:20 |
| CS1870 |  | AD BRAAK STAGE II |  | 97 |  | FEMALE |  | 10:30 |
| CS1357 |  | AD BRAAK STAGE II |  | 79 |  | FEMALE |  | 13:57 |
| CS1109 |  | AD BRAAK STAGE II |  | 75 |  | FEMALE |  | 16:20 |
| CS1431 |  | AD BRAAK STAGE II |  | 95 |  | FEMALE |  | 20:00 |
| CS1112 |  | AD BRAAK STAGE III |  | 83 |  | FEMALE |  | 07:30 |
| CS1144 |  | AD BRAAK STAGE III |  | 73 |  | MALE |  | 04:20 |
| CS1759 |  | AD BRAAK STAGE III |  | 74 |  | MALE |  | 09:00 |
| CS0754 |  | AD BRAAK STAGE III |  | 87 |  | MALE |  | 02:30 |
| CS1057 |  | AD BRAAK STAGE III |  | 83 |  | FEMALE |  | 04:30 |
| CS1247 |  | AD BRAAK STAGE III |  | 80 |  | FEMALE |  | 08:00 |
| CS1194 |  | AD BRAAK STAGE III |  | 92 |  | FEMALE |  | 05:10 |
| CS1085 |  | AD BRAAK STAGE III |  | 78 |  | FEMALE |  | 08:15 |
| CS0497 |  | AD BRAAK STAGE IV |  | 82 |  | MALE |  | 02:30 |
| CS0774 |  | AD BRAAK STAGE IV |  | 75 |  | MALE |  | 10:00 |
| CS1403 |  | AD BRAAK STAGE IV |  | 85 |  | FEMALE |  | 07:00 |
| CS0957 |  | AD BRAAK STAGE IV |  | 85 |  | FEMALE |  | 04:30 |
| CS0963 |  | AD BRAAK STAGE IV |  | 83 |  | FEMALE |  | 04:30 |
| CS1146 |  | AD BRAAK STAGE IV |  | 86 |  | FEMALE |  | 05:30 |
| CS1043 |  | AD BRAAK STAGE IV |  | 87 |  | FEMALE |  | 05:30 |
| CS0948 |  | AD BRAAK STAGE IV |  | 79 |  | MALE |  | 05:30 |
| CS1255 |  | AD BRAAK STAGE IV |  | 89 |  | MALE |  | 07:00 |
| CS0597 |  | AD BRAAK STAGE V |  | 75 |  | FEMALE |  | 08:30 |
| CS0676 |  | AD BRAAK STAGE V |  | 78 |  | MALE |  | 05:30 |
| CS0723 |  | AD BRAAK STAGE V |  | 89 |  | FEMALE |  | 08:50 |
| CS0836 |  | AD BRAAK STAGE V |  | 87 |  | FEMALE |  | 03:00 |
| CS0916 |  | AD BRAAK STAGE V |  | 85 |  | FEMALE |  | 04:00 |
| CS0997 |  | AD BRAAK STAGE V |  | 81 |  | FEMALE |  | 05:30 |
| CS1126 |  | AD BRAAK STAGE V |  | 73 |  | MALE |  | 06:30 |
| CS1230 |  | AD BRAAK STAGE V |  | 79 |  | MALE |  | 04:15 |
| CS1262 |  | AD BRAAK STAGE V |  | 78 |  | MALE |  | 07:20 |
| CS1488 |  | AD BRAAK STAGE V |  | 77 |  | MALE |  | 07:30 |
| CS1051 |  | AD BRAAK STAGE VI |  | 76 |  | MALE |  | 06:30 |
| CS1145 |  | AD BRAAK STAGE VI |  | 86 |  | MALE |  | 05:45 |
| CS1556 |  | AD BRAAK STAGE VI |  | 77 |  | MALE |  | 05:30 |
| CS0869 |  | AD BRAAK STAGE VI |  | 89 |  | FEMALE |  | 07:50 |
| CS0430 |  | AD BRAAK STAGE VI |  | 77 |  | FEMALE |  | 06:15 |
| CS1161 |  | AD BRAAK STAGE VI |  | 89 |  | FEMALE |  | 04:15 |
| CS0729 |  | AD BRAAK STAGE VI |  | 77 |  | FEMALE |  | 04:30 |
| CS0785 |  | AD BRAAK STAGE VI |  | 82 |  | MALE |  | 05:00 |
| CS0807 |  | AD BRAAK STAGE VI |  | 75 |  | MALE |  | 08:15 |
| CS0941 |  | AD BRAAK STAGE VI |  | 73 |  | MALE |  | 05:00 |
| A/P = Pahological/Anatomical. PM = Postmortem | | | | | | | | |
