# Supplementary material for: Lipidomic Analysis of Human Plasma and Hippocampus Across Alzheimer’s Progression and Preclinical 5xFAD Mouse Model
Source: Mol Neurobiol. 2026 Apr 13;63(1):561. doi: 10.1007/s12035-026-05849-1 (PMC13076374; doi:10.1007/s12035-026-05849-1)
Supplement: Supplementary file 7 — (29.7 KB DOCX) [file 12035_2026_5849_MOESM7_ESM.docx]

**Table S2.**  Lipid classes detected in Braak I-II, Braak III-IV, and Braak V-VI hippocampal samples.

| Class | Species detected |
| --- | --- |
| PC | [30:0]; [32:1]; [32:0]; [34:2]; [34:1]; [34:0]; [36:4]; [36:3]; [36:2]; [36:1]; [38:6]; [38:5]; [38:4]; [38:3]; [38:2]; [38:1]; [40:7]; [40:6]; [40:5]; [40:4]; [44:2] |
| PC-O | [32:1]; [32:0]; [34:5]; [34:4]; [34:3]; [34:2]; [34:1]; [34:0]; [36:6]; [36:5]; [36:4]; [36:3]; [36:2]; [36:1]; [38:6]; [38:5]; [38:4]; [40:6]; [40:5]; [40:4] |
| LPC | [16:0]; [18:1]; [18:0]; [20:4] |
| PI | [34:2]; [34:1]; [36:4]; [36:3]; [36:2]; [36:1]; [38:6]; [38:5]; [38:4]; [38:3]; [40:7]; [40:6]; [40:5]; [40:4] |
| PE-P | [34:2]; [34:1]; [34:0]; [36:4]; [36:3]; [36:2]; [36:1]; [38:6]; [38:5]; [38:4]; [38:3]; [38:2]; [38:1]; [40:7]; [40:6]; [40:5]; [40:4]; [40:3]; [40:2]; [42:6]; [42:5]; [42:4] |
| LPC | [16:0]; [18:1]; [18:0]; [20:4] |
| LPE | [16:0]; [18:1]; [18:0]; [20:4]; [20:1]; [22:6]; [22:5]; [22:4] |
| LPE-P | [18:1]; [18:0] |
| PE | [34:1]; [34:0]; [36:4]; [36:2]; [36:1]; [38:6]; [38:5]; [38:4]; [38:3]; [38:2]; [38:1]; [40:6]; [40:5]; [40:4]; [40:3] |
| LPI | [16:0]; [18:1]; [18:0]; [20:4]; [20:3] |
| LPS | [18:1]; [18:0]; [22:6]; [22:5]; [22:4] |
| PS | [34:1]; [36:2]; [36:1]; [38:5]; [38:4]; [38:3]; [38:2]; [38:1]; [40:7]; [40:6]; [40:4]; [40:3]; [40:2]; [40:1]; [42:5]; [42:4]; [42:3]; [42:2] |
| LPG | [16:0]; [18:2]; [18:1]; [20:4]; [20:3]; [22:6]; [22:5] |
| PG | [32:1]; [32:0]; [34:2]; [34:1]; [36:4]; [36:3]; [36:2]; [38:6]; [38:5]; [38:4]; [40:8]; [40:7]; [40:6]; [42:10]; [42:9]; [44:12]; [44:11] |
| PA | [32:1]; [32:0]; [34:2]; [34:1]; [34:0]; [36:2]; [36:1]; [38:4]; [38:2]; [38:1]; [40:6] |
| LCL | [52:4]; [52:3]; [52:2]; [54:6]; [54:5]; [54:4]; [54:3]; [56:7]; [56:6]; [58:9]; [58:8] |
| CL | [68:5]; [68:4]; [68:3]; [68:2]; [70:7]; [70:6]; [70:5]; [70:4]; [70:3]; [72:9]; [72:8]; [72:7]; [72:6]; [72:5]; [72:4]; [74:11]; [74:10]; [74:9]; [74:8]; [74:7]; [74:6]; [76:12]; [76:11]; [76:10]; [76:9]; [76:8]; [78:13]; [78:12]; [78:11]; [78:10]; [80:15]; [80:14] |
| SM | [34:1:2]; [36:2:2]; [36:1:2]; [38:1:2]; [40:2:2]; [40:1:2]; [41:2:2]; [41:1:2]; [42:3:2]; [42:2:2]; [42:1:2]; [43:2:2]; [43:1:2]; [44:2:2] |
| Cer | [34:1:2]; [36:2:2]; [36:1:2]; [36:0:2]; [37:1:2]; [38:2:2]; [38:1:2]; [40:2:2]; [40:1:2]; [41:1:2]; [42:3:2]; [42:2:2]; [42:1:2]; [43:2:2]; [43:1:2]; [44:2:2] |
| HexCer | [36:2:2]; [36:1:2]; [36:1:3]; [38:1:2]; [38:2:3]; [40:1:2]; [40:2:3]; [41:1:2]; [40:1:3]; [42:2:2]; [41:2:3]; [42:1:2]; [41:1:3]; [42:3:3]; [43:2:2]; [42:2:3]; [43:1:2]; [42:1:3]; [44:2:2]; [43:2:3]; [44:1:2]; [44:2:3] |
| Sulf | [36:1:2]; [40:2:2]; [40:1:2]; [41:2:2]; [41:1:2]; [40:1:3]; [42:2:2]; [41:2:3]; [42:1:2]; [41:1:3]; [43:2:2]; [42:2:3]; [43:1:2]; [42:1:3]; [44:2:2]; [43:2:3]; [44:1:2]; [43:1:3]; [44:2:3] |
| GM1 | [36:1:2]; [38:1:2] |
| GD1 | [36:2:2]; [36:1:2]; [38:2:2]; [38:1:2]; [38:2:3]; [40:1:2]; [40:2:3]; [42:2:2] |
| GD3 | [36:1:2]; [38:1:2] |
| GT1 | [36:1:2]; [38:1:2]; [40:2:3] |
| DG | [34:1]; [36:4]; [36:3]; [36:2]; [38:4]; [38:3] |
| FFA | [18:2]; [20:4]; [20:3]; [20:2]; [20:1]; [22:6]; [22:5]; [22:4] |
| Carn | [0:0] |
| AcCar | [2:0] |
