# Supplementary material for: Lipidomic Analysis of Human Plasma and Hippocampus Across Alzheimer’s Progression and Preclinical 5xFAD Mouse Model
Source: Mol Neurobiol. 2026 Apr 13;63(1):561. doi: 10.1007/s12035-026-05849-1 (PMC13076374; doi:10.1007/s12035-026-05849-1)
Supplement: Supplementary file 8 — (16.7 KB DOCX) [file 12035_2026_5849_MOESM8_ESM.docx]

| **Table S3. Human plasma sample list** | | | | | | |
| --- | --- | --- | --- | --- | --- | --- |
| **ID NUMBER** |  | **CLINICAL DIAGNOSIS** |  | **AGE** |  | **SEX** |
| 1048195891 |  | CONTROL |  | 62 |  | MALE |
| 1048194965 |  | CONTROL |  | 68 |  | MALE |
| 1048193367 |  | CONTROL |  | 68 |  | FEMALE |
| 1048193500 |  | CONTROL |  | 70 |  | FEMALE |
| 1048193524 |  | CONTROL |  | 71 |  | MALE |
| 1048193523 |  | CONTROL |  | 60 |  | FEMALE |
| 1048193497 |  | CONTROL |  | 62 |  | MALE |
| 1048196733 |  | MCI |  | 70 |  | MALE |
| 1048195744 |  | MCI |  | 70 |  | FEMALE |
| 1044180262 |  | MCI |  | 64 |  | MALE |
| 1044117184 |  | MCI |  | 77 |  | MALE |
| 1044174155 |  | MCI |  | 68 |  | FEMALE |
| 1044174140 |  | MCI |  | 71 |  | MALE |
| 1048195937 |  | AD LIKE DEMENTIA |  | 60 |  | FEMALE |
| 1048195898 |  | AD LIKE DEMENTIA |  | 59 |  | FEMALE |
| 1048193463 |  | AD LIKE DEMENTIA |  | 63 |  | FEMALE |
| 1044175878 |  | AD LIKE DEMENTIA |  | 55 |  | FEMALE |
| 1044177395 |  | AD LIKE DEMENTIA |  | 59 |  | FEMALE |
| AAA1924565 |  | AD LIKE DEMENTIA |  | 59 |  | MALE |
| AAA1919142 |  | AD LIKE DEMENTIA |  | 66 |  | FEMALE |
| MCI = Mild Cognitive Impairment | | | | | | |
