# Supplementary material for: Lipidomic Analysis of Human Plasma and Hippocampus Across Alzheimer’s Progression and Preclinical 5xFAD Mouse Model
Source: Mol Neurobiol. 2026 Apr 13;63(1):561. doi: 10.1007/s12035-026-05849-1 (PMC13076374; doi:10.1007/s12035-026-05849-1)
Supplement: Supplementary file 9 — (15.9 KB DOCX) [file 12035_2026_5849_MOESM9_ESM.docx]

**Table S4**. Lipid classes detected in healthy, MCI and AD plasma samples.

| Class | Species detected |
| --- | --- |
| PC | PC [32:1]; [32:0]; [34:3]; [34:2]; [34:1]; [36:5]; [36:4]; [36:3]; [36:2]; [36:1]; [38:6]; [38:5]; [38:4]; [38:3]; [40:7]; [40:6]; [40:5] |
| PC-O | PC-O [32:1]; [32:0]; [34:3]; [34:2]; [34:1]; [36:5]; [36:4]; [36:3]; [36:2]; [38:6]; [38:5]; [38:4]; [38:3]; [40:6]; [40:5]; [40:4]; [42:6]; [42:5] |
| PI | PI [34:2]; [34:1]; [36:4]; [36:3]; [36:2]; [36:1]; [38:5]; [38:4]; [38:3]; [40:6] |
| PE-Pl | PE-Pl [34:2]; [34:1]; [36:5]; [36:4]; [36:3]; [36:2]; [36:1]; [38:6]; [38:5]; [38:4]; [38:3]; [40:7]; [40:6]; [40:5]; [40:4] |
| LPC | LPC [18:2]; [18:1]; [18:0]; [20:4]; [20:3]; [22:6] |
| LPE | LPE [16:0]; [18:2]; [18:1]; [18:0]; [20:4]; [20:2]; [20:1]; [22:6]; [22:4] |
| PE | PE [34:2]; [34:1]; [36:4]; [36:3]; [36:2]; [36:1]; [38:6]; [38:5]; [38:4]; [38:3]; [38:2]; [40:6]; [40:5]; [40:4]; [40:3] |
| LPI | LPI [18:0]; [20:4] |
| SM | SM [32:1:2]; [33:1:2]; [34:2:2]; [34:1:2]; [34:0:2]; [35:1:2]; [36:3:2]; [36:2:2]; [36:1:2]; [38:2:2]; [38:1:2]; [39:1:2]; [40:2:2]; [40:1:2]; [41:2:2]; [41:1:2]; [42:3:2]; [42:2:2]; [42:1:2]; [43:2:2]; [43:1:2] |
| Cer | Cer [34:1:2]; [36:1:2]; [38:1:2]; [38:0:2]; [40:2:2]; [40:1:2]; [40:0:2]; [41:1:2]; [42:3:2]; [42:2:2]; [42:1:2]; [42:0:2]; [42:1:3]; [43:1:2]; [43:2:2] |
| HexCer | HexCer [34:1:2]; [40:1:2]; [42:2:2]; [41:2:3]; [42:1:2] |
| GM3 | GM3 [34:1:2]; [42:2:2] |
| DG | DG [34:2]; [34:1]; [36:4]; [36:3]; [36:2] |
| TG | TG [48:2]; [48:1]; [50:3]; [50:2]; [50:1]; [52:5]; [52:4]; [52:3]; [52:2]; [54:7]; [54:6]; [54:5]; [54:4]; [54:3]; [54:2]; [56:8]; [56:7]; [56:6]; [56:5]; [58:8]; [58:7] |
| CE | CE [16:1]; [16:0]; [18:3]; [18:2]; [18:1]; [20:5]; [20:4]; [20:3]; [22:6] |
| FFA | FFA [18:3]; [20:4]; [20:3]; [20:2]; [22:6]; [22:5]; [22:4] |
| Carn | Carn [0:0] |
| AcCar | AcCar [2:0]; [3:0]; [4:0]; [16:0]; [18:1]; [18:0] |
