# Supplementary material for: Lipidomic Analysis of Human Plasma and Hippocampus Across Alzheimer’s Progression and Preclinical 5xFAD Mouse Model
Source: Mol Neurobiol. 2026 Apr 13;63(1):561. doi: 10.1007/s12035-026-05849-1 (PMC13076374; doi:10.1007/s12035-026-05849-1)
Supplement: Supplementary file 12 — (23.2 KB DOCX) [file 12035_2026_5849_MOESM12_ESM.docx]

**Human hippocampus standard mixtures**

| **Lipid Class** | **Ion format** | **Internal standard (IS)** | **IS conc in infusion solvent (fmol/uL)** |
| --- | --- | --- | --- |
| PC, PC-O, LPC | [M+H]^+^ | PC(15:0/18:1d7) | 480 |
| PE, PE-P, LPE | [M−H]^−^ | PE(15:0/18:1d7) | 170 |
| PI, LPI | [M−H]^−^ | PI(15:0/18:1d7) | 119 |
| PS, LPS | [M−H]^−^ | PS(15:0/18:1d7) | 129 |
| PG/BMP, LPG | [M−H]^−^ | PG(15:0/18:1d7) | 26 |
| PA | [M−H]^−^ | PA(15:0/18:1d7) | 29 |
| CL, LCL | [M−2H]^2−^ | CL(56:4) | 16 |
| SM | [M+H]^+^ | SM(36:2:2d9) | 109 |
| Cer | [M+Cl]^−^ | Cer(35:1:2) | 36 |
| HexCer | [M+Cl]^−^ | GluCer(30:1:2) | 31 |
| LacCer, Gb3 | [M+Cl]^−^ | LacCer(35:1:2) | 23 |
| GM3 | [M−H]^−^ | GM3(d18:1/D3-18:0) | 67 |
| GM1 (GD, GT) | [M−H+Cl]^2−^ | GM1(d18:1/D3-18:0) | 103 |
| Sulf | [M−H]^−^ | Sulf(30:1:2) | 27 |
| DG | [M+NH_4_]^+^ | DG(15:0/18:1d7) | 34 |
| TG | [M+NH_4_]^+^ | TG(15:0/18:1d7/15:0) | 124 |
| CE | [M+NH_4_]^+^ | Chold7E(16:1) | 153 |
| FFA | [M−H]^−^ | FFA(19:0) | 135 |
| Carn | [M+H]^+^ | Carnitine d9 | 92 |
| AcCar | [M+H]^+^ | AcCar(2:0d3) | 23 |

**Human plasma standard mixtures**

| **Lipid Class** | **Ion format** | **Internal standard (IS)** | **IS conc in infusion solvent (fmol/uL)** |
| --- | --- | --- | --- |
| PC, PC-O, LPC | [M+H]^+^ | PC(15:0/18:1d7) | 843 |
| PE, PE-P, LPE | [M−H]^−^ | PE(15:0/18:1d7) | 151 |
| PI, LPI | [M−H]^−^ | PI(15:0/18:1d7) | 126 |
| PS, LPS | [M−H]^−^ | PS(15:0/18:1d7) | 23 |
| PG/BMP, LPG | [M−H]^−^ | PG(15:0/18:1d7) | 23 |
| PA | [M−H]^−^ | PA(15:0/18:1d7) | 26 |
| SM | [M+H]^+^ | SM(36:2:2d9) | 158 |
| Cer | [M+Cl]^−^ | Cer(35:1:2) | 32 |
| HexCer | [M+Cl]^−^ | GluCer(30:1:2) | 28 |
| GM3 | [M−H]^−^ | GM3(d18:1/D3-18:0) | 15 |
| DG | [M+NH_4_]^+^ | DG(15:0/18:1d7) | 36 |
| TG | [M+NH_4_]^+^ | TG(15:0/18:1d7/15:0) | 313 |
| CE | [M+NH_4_]^+^ | Chold7E(16:1) | 3218 |
| FFA | [M−H]^−^ | FFA(19:0) | 239 |
| Carn | [M+H]^+^ | Carnitine d9 | 48 |
| AcCar | [M+H]^+^ | AcCar(2:0d3) | 12 |

**Mouse hippocampus standard mixtures**

| **Lipid Class** | **Ion format** | **Internal standard (IS)** | **IS conc in infusion solvent (fmol/uL)** |
| --- | --- | --- | --- |
| PC, PC-O, LPC | [M+H]^+^ | PC(15:0/18:1d7) | 480 |
| PE, PE-P, LPE | [M−H]^−^ | PE(15:0/18:1d7) | 170 |
| PI, LPI | [M−H]^−^ | PI(15:0/18:1d7) | 119 |
| PS, LPS | [M−H]^−^ | PS(15:0/18:1d7) | 129 |
| PG/BMP, LPG | [M−H]^−^ | PG(15:0/18:1d7) | 26 |
| PA | [M−H]^−^ | PA(15:0/18:1d7) | 29 |
| CL, LCL | [M−2H]^2−^ | CL(56:4) | 16 |
| SM | [M+H]^+^ | SM(36:2:2d9) | 109 |
| Cer | [M+Cl]^−^ | Cer(35:1:2) | 36 |
| HexCer | [M+Cl]^−^ | GluCer(30:1:2) | 31 |
| LacCer, Gb3 | [M+Cl]^−^ | LacCer(35:1:2) | 23 |
| GM3 | [M−H]^−^ | GM3(d18:1/D3-18:0) | 67 |
| GM1 (GD, GT) | [M−H+Cl]^2−^ | GM1(d18:1/D3-18:0) | 103 |
| Sulf | [M−H]^−^ | Sulf(30:1:2) | 27 |
| DG | [M+NH_4_]^+^ | DG(15:0/18:1d7) | 34 |
| TG | [M+NH_4_]^+^ | TG(15:0/18:1d7/15:0) | 124 |
| CE | [M+NH_4_]^+^ | Chold7E(16:1) | 153 |
| FFA | [M−H]^−^ | FFA(19:0) | 135 |
| Carn | [M+H]^+^ | Carnitine d9 | 92 |
| AcCar | [M+H]^+^ | AcCar(2:0d3) | 23 |

**Mouse plasma standard mixtures**

| **Lipid Class** | **Ion format** | **Internal standard (IS)** | **IS conc in infusion solvent (fmol/uL)** |
| --- | --- | --- | --- |
| PC, PC-O, LPC | [M+H]^+^ | PC(15:0/18:1d7) | 843 |
| PE, PE-P, LPE | [M−H]^−^ | PE(15:0/18:1d7) | 151 |
| PI, LPI | [M−H]^−^ | PI(15:0/18:1d7) | 126 |
| PS, LPS | [M−H]^−^ | PS(15:0/18:1d7) | 23 |
| PG/BMP, LPG | [M−H]^−^ | PG(15:0/18:1d7) | 23 |
| PA | [M−H]^−^ | PA(15:0/18:1d7) | 26 |
| SM | [M+H]^+^ | SM(36:2:2d9) | 158 |
| Cer | [M+Cl]^−^ | Cer(35:1:2) | 32 |
| HexCer | [M+Cl]^−^ | GluCer(30:1:2) | 28 |
| GM3 | [M−H]^−^ | GM3(d18:1/D3-18:0) | 15 |
| DG | [M+NH_4_]^+^ | DG(15:0/18:1d7) | 36 |
| TG | [M+NH_4_]^+^ | TG(15:0/18:1d7/15:0) | 313 |
| CE | [M+NH_4_]^+^ | Chold7E(16:1) | 3218 |
| FFA | [M−H]^−^ | FFA(19:0) | 239 |
| Carn | [M+H]^+^ | Carnitine d9 | 48 |
| AcCar | [M+H]^+^ | AcCar(2:0d3) | 12 |
